# Evolutionary Paths of the cAMP-Dependent Protein Kinase (PKA) Catalytic Subunits

**DOI:** 10.1371/journal.pone.0060935

**Published:** 2013-04-12

**Authors:** Kristoffer Søberg, Tore Jahnsen, Torbjørn Rognes, Bjørn S. Skålhegg, Jon K. Laerdahl

**Affiliations:** 1 Department of Nutrition, Institute of Basic Medical Sciences, University of Oslo, Oslo, Norway; 2 Department of Biochemistry, Institute of Basic Medical Sciences, University of Oslo, Oslo, Norway; 3 Department of Informatics, University of Oslo, Oslo, Norway; 4 Centre for Molecular Biology and Neuroscience (CMBN), Department of Microbiology, Oslo University Hospital Rikshospitalet, Oslo, Norway; 5 Bioinformatics Core Facility, Department of Informatics, University of Oslo, Oslo, Norway; Indian Institute of Science, India

## Abstract

3′,5′-cyclic adenosine monophosphate (cAMP) dependent protein kinase or protein kinase A (PKA) has served as a prototype for the large family of protein kinases that are crucially important for signal transduction in eukaryotic cells. The PKA catalytic subunits Cα and Cβ, encoded by the two genes *PRKACA* and *PRKACB*, respectively, are among the best understood and characterized human kinases. Here we have studied the evolution of this gene family in chordates, arthropods, mollusks and other animals employing probabilistic methods and show that Cα and Cβ arose by duplication of an ancestral PKA catalytic subunit in a common ancestor of vertebrates. The two genes have subsequently been duplicated in teleost fishes. The evolution of the *PRKACG* retroposon in simians was also investigated. Although the degree of sequence conservation in the PKA Cα/Cβ kinase family is exceptionally high, a small set of signature residues defining Cα and Cβ subfamilies were identified. These conserved residues might be important for functions that are unique to the Cα or Cβ clades. This study also provides a good example of a seemingly simple phylogenetic problem which, due to a very high degree of sequence conservation and corresponding weak phylogenetic signals, combined with problematic nonphylogenetic signals, is nontrivial for state-of-the-art probabilistic phylogenetic methods.

## Introduction

Protein kinases are enzymes that catalyze the transfer of a phosphate group from adenosine 5′-triphosphate (ATP) to a serine, threonine, tyrosine or other residue on a substrate. Most eukaryotic protein kinases derive from a common ancestor kinase, and share the same core catalytic domain [1].

PKA (EC 2.7.11.11) is a serine/threonine kinase which is ubiquitously expressed in the human body. It is involved in many intracellular signaling events, and its function, specificity, and downstream effects depend on factors such as subcellular localization, expression of a number of isoforms and physio-chemical features [2], [3]. The inactive form of PKA is a heterotetrameric holoenzyme consisting of a regulatory (R) subunit dimer binding to two catalytic (C) subunits [4]. During activation, cAMP binds cooperatively to two sites termed A and B on each R subunit. In the inactive holoenzyme, only the B site is exposed and available for cAMP binding. When occupied, this enhances the binding of cAMP to the A site which leads to an intramolecular conformational change and the release of the R subunit dimer. The two C monomers are then free to phosphorylate relevant C substrates in the cytosol and nucleus [5]–[7]. Thus, a major function of the R subunit is to inhibit the phosphotransferase activity of the C subunits through direct interactions.

Several variants of the C and R subunits have been identified in human cells. Four R subunits designated RIα, RIβ, RIIα, and RIIβ are transcribed from separate genes [8]. PKA holoenzymes containing RI and RII subunits are designated PKA type I and II, respectively [9], [10]. Protein-protein interactions and organization of signal transduction pathways is required to obtain specificity in space and time. Protein kinases are localized to the relevant subcellular sites through anchoring, scaffolding and adapter protein activity. Major organizers of the cAMP signaling pathway are the A kinase anchoring proteins (AKAPs), which both function as scaffolding proteins and attach PKA to subcellular structures [11], [12]. Initially, AKAPs were shown to interact with the RII subunits [13]. Later, dual specific AKAPs binding both RII and RI, as well as AKAPs binding only RI, have been identified, demonstrating that both PKA type I and II may be tethered to subcellular compartments in the cell [14]–[16].

**Figure 1:**
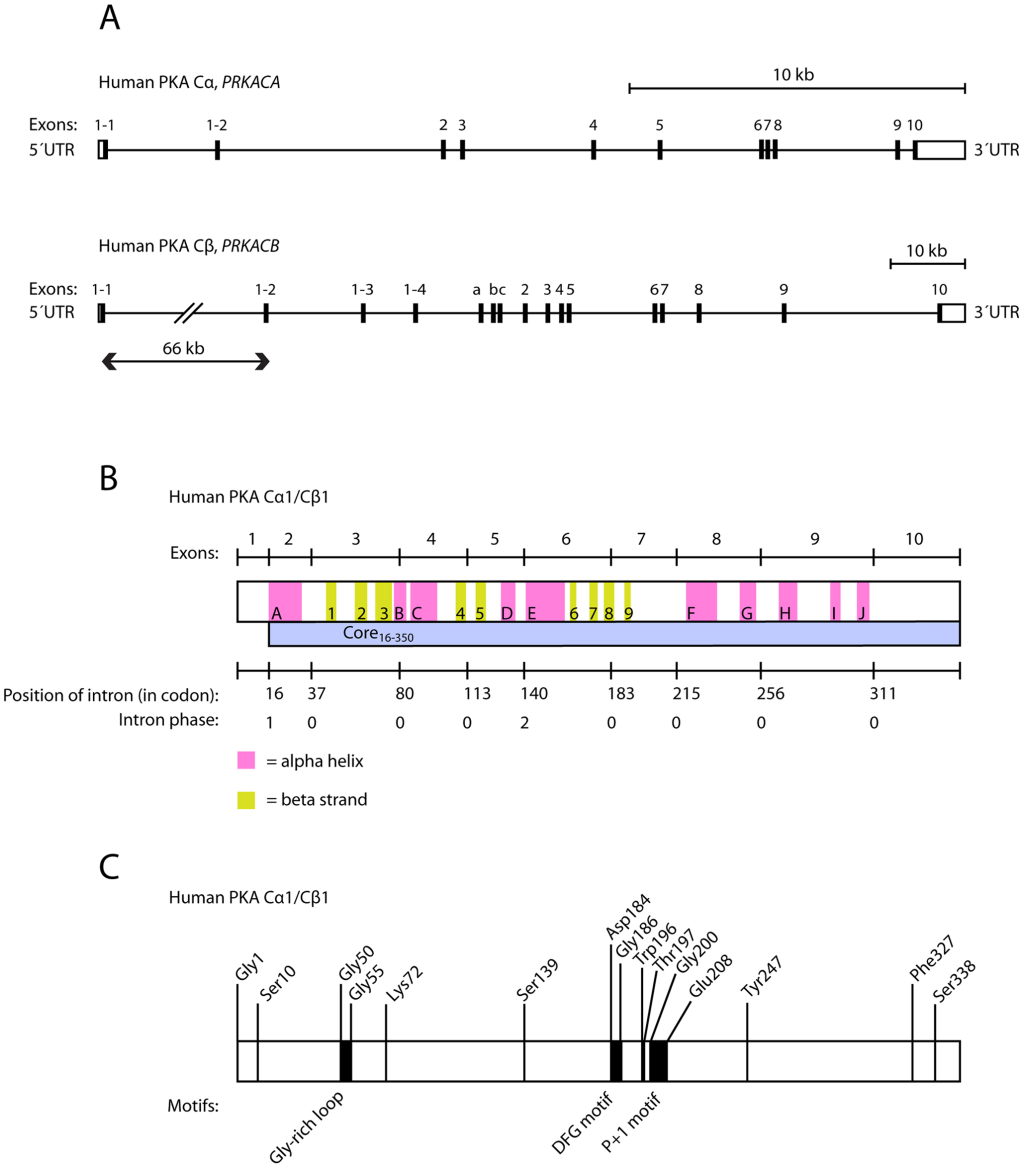
Gene structure and overview of the human PKA catalytic subunits Cα and Cβ encoded by the *PRKACA* and *PRKACB* genes, respectively. **A** Exons, introns and 3′ and 5′ untranslated regions (UTRs) of the PKA catalytic subunit genes are shown. The human Cα gene (*PRKACA*) is located at chromosome 19p13.1 (reverse strand) and has a length of approximately 26 000 nucleotides (nt). Alternative transcription start sites give rise to two splice variants known as Cα1 and Cα2 (formerly known as CαS). Both splice variants comprise exons 2 to 10 and in addition a 5′ exon 1–1 or 1–2 in Cα1 and Cα2, respectively. The human Cβ gene (*PRKACB*) is located at chromosome 1p31.1 (forward strand), with a length of approximately 160 000 nt. Alternative splicing of exons 1–1, 1–2, 1–3 and 1–4 give rise to the splice variants Cβ1, Cβ2, Cβ3 and Cβ4, respectively. In addition, three short exons, a, b and c, have been shown to be included in the transcript in various combinations. All known enzymatically active isoforms of Cβ comprise exons 2 to 10. **B** Human PKA Cα1 consists of ten α-helix and nine β-strand secondary structure elements [76]. The figure (middle box) gives the location of α-helices (pink, A–J) and β-strands (yellow, 1–9) relative to the ten exons and 351 encoded codons of the Cα1 isoform. The locations of the boundaries between exons are given on the upper line. The codons corresponding to the nine introns, as well as their intron phases, are given on the lower line. The intron phase is defined as the position of the intron within a codon, with phase 0, 1, or 2 lying before the first base, after the first base, or after the second base, respectively. Human PKA Cβ1 has the same length as Cα1, and the two proteins are differing at only 25 amino acid positions (92.9% sequence identity), strongly suggesting that the overall 3D structures, including secondary structure elements, are close to identical. The position and intron phases for the nine introns are also conserved between human Cα1 and Cβ1. The sequence segment corresponding to exons 2–10, termed Core_16–350_, is shown as a blue bar. **C** The function of selected important residues and motifs in human PKA catalytic subunits has previously been elucidated in the literature. All listed residues, and their numbering, are identical in Cα1 and Cβ1, but the research describing these residues and motifs has mainly been performed on Cα. Numbering of amino acids is given for mature Cα1 and Cβ1 (with N-terminal Met removed), both encoding 350 residues. Gly1 is found to be posttranslationally modified by myristoylation [77]. Ser10, Ser139 and Ser338 are well characterized phosphorylation sites [78], [79]. The Gly-rich loop (Gly50–Gly55) plays an important role in phosphoryl transfer [80]–[82]. Lys72 and Asp184 are crucial for ATP and Mg^2+^ binding in the active site [81] and the DFG motif (Asp184–Gly186) is conserved in most kinases. The conformation of the motif is critical for the functional state of the kinase [83], [84]. Phe327 is the only residue outside of the kinase core binding to the adenine of ATP [82]. Trp196 is an essential residue for R subunit binding and phosphorylation of Thr197 is necessary for the enzyme to assume the active conformation, thereby facilitating catalysis as well as R subunit binding [5], [85]. The hydrophobic P+1 motif (Gly200–Glu208) is important for the structure of the enzyme, as well as for substrate recognition [80], [86]. Tyr247 competes with cAMP for R subunit binding [5].

Five different human C subunit genes have been identified; *PRKACA*, *PRKACB*, *PRKACG*, *PRKX*, and *PRKY* [2], [17]–[19], all within the AGC group of kinases, which contains the cyclic-nucleotide-dependent family (PKA and PKG), the protein kinase C family, the β-adrenergic receptor kinase, the ribosomal S6 family and some other relatives of these kinases [1], [20]. Three of the human C subunit genes, *PRKACA*, *PRKACB*, and *PRKX*, have been demonstrated to be transcribed and translated into functional protein kinases, termed PKA Cα, PKA Cβ, and PRKX, respectively. Cα exhibits two splice variants, Cα1 [21] and Cα2 [22]–[24], by employing two alternative 5′ exons in *PRKACA* (Fig. 1A). Whereas Cα1 is ubiquitously expressed in man, Cα2 is exclusively expressed in the sperm cell and has been shown to be essential for sperm motility and fertilization [24]–[27]. A number of isoforms of human *PRKACB* have been identified with alternative splicing of exons 5′ of exon 2 (Fig. 1A), encoding at least the following proteins: Cβ1, Cβ2, Cβ3, Cβ4, Cβ3ab, Cβ3b, Cβ3abc, Cβ4ab, Cβ4b, and Cβ4abc [28]–[33]. In addition, Cβ variants formed by skipping of exon 4 are expressed in the brain of higher primates. These C subunits bind the R subunit in a cAMP-independent fashion [34].

PRKX, which is a protein kinase encoded from the X-chromosome, also binds RIα in a cAMP sensitive fashion [19]. PRKX and PRKY are 94% identical and have unknown functions [18], [20]. *PRKACG* is a retroposon lacking introns and may be a pseudogene [35]. Whereas mRNA from *PRKACG* is solely, but ubiquitously, transcribed in the testis [17], the corresponding protein Cγ has never been identified. *In vitro* experiments on expressed Cγ have revealed a functional kinase with variant-specific properties. The Cγ protein kinase is, in contrast to Cα, and most probably Cβ, not inhibited by the Protein Kinase Inhibitor (PKI), and it requires higher concentrations of cAMP for dissociation from PKA type I holoenzymes [36], [37]. The *in vivo* function of PKA Cγ, if the protein exists, remains to be elucidated.

The *PRKACA* and *PRKACB* genes share identical positions and intron phases for all nine introns, and are very likely the result of a gene duplication event (Fig. 1B). Human PKA Cα1 and Cβ1 have the same length (350 residues) and share 93% sequence identity. Many studies have elucidated the importance and function of a number of residues and short sequence segments in these kinases, and some of these are briefly summarized in Fig. 1C. The sequence identity between human PKA Cα vs. Cγ and PRKX is, 82% and 54%, respectively, and between Cα/Cβ and other human kinases below 50%. PKA C kinases in invertebrate metazoa have sequence identity with human Cα/Cβ above 77% (see below), confirming that the PKA Cα/Cβ-like kinases, not including PRKX, builds a unique and compact clade/family of kinases within the AGC-group.

A previous study explored the evolution of the R subunits of PKA [38]. Based on a multiple sequence alignment (MSA) of the most conserved region in the R subunit sequences, the phosphate-binding cassette, the authors proposed a new classification of the R subunits in place of a classification based on physiochemical properties. They also identified a signature sequence that characterizes the R subunit genes, and identified type- and subtype-specific residues. The same focused analysis of the C subunits of PKA is to our knowledge lacking. In order to get a comprehensive overview of all the known PKA genes and obtain insight into the essential residues of these proteins, we have collected, compared and performed an extensive analysis of PKA C subunit sequences from a large number of bilaterian animal species. The homologous genes *PRKACA* and *PRKACB* constitute a unique clade of kinases and their protein products are the main sources of PKA activity in the cell. Therefore, we focused our analysis on the Cα/Cβ-like kinases, including Cγ, but not the more remotely related kinases PRKX and PRKY. The main focus of this study has been on elucidating the phylogeny of vertebrate and other chordate PKA Cα/Cβ homologs, while orthologous sequences from mollusks and arthropods were included mainly to serve as outgroups in the phylogenetic analysis. We show that an ancestor C subunit was duplicated around the time of the evolution of the first vertebrate species, giving rise to the paralogous genes encoding Cα and Cβ. Further analysis on the Cα/Cβ homologs revealed signature sequences characteristic of the two paralogs. Comparison of Cα and Cβ sequences gave insight into molecular differences and possible mechanisms that may determine functional differences between these two prototype protein kinases.

## Materials and Methods

### Sequences of PKA Cα/Cβ Homologs

Homologous sequences of human PKA Cα/Cβ were obtained from the UniProt [39] and NCBI [40] database resources and from the Ensembl project [41] as described in Materials and Methods S1. Standard BLAST sequence searching algorithms were employed [42] and only sequences with sequence identity (protein level) compared with human PKA Cα or Cβ above 75% were included in the dataset for further analysis. The final dataset comprised 41 sequences from placental mammals, including 18 from primates, 5 marsupial sequences, 35 sequences from non-mammalian vertebrates, and 15 from invertebrates (See Materials and Methods S1 and Table S1). Both nucleotide and protein sequences, one single splice variant for each gene, were stored in FASTA format for the total 96 sequences. 81 sequences appear to be full-length, comprising exons 2 to 10 and in addition a 5′ exon chosen to correspond to human Cα1/Cβ1 when possible. Of the incomplete sequences, 8 were missing only the 5′ exon. In addition, one *Petromyzon marinus* sequence was missing exons 1, 7, 9, and 10, and the two sequences from *Macropus eugenii* were missing exons 1 and 9, and part of the 3′ end, respectively. Finally, the four cartilaginous fish sequences only contain fragments of the full-length sequence.

The sequences were aligned with MUSCLE [43] and the MSAs were viewed and edited with Jalview [44] (See Figure S1). All analysis was subsequently based on MSAs corresponding to exons 2–10 of human PKA Cα1/Cβ1 (*i.e.* residues 16–350, Fig. 1B) unless otherwise stated, and columns of the MSAs containing gaps were deleted. PHYLIP and NEXUS format files were generated from the FASTA files with a dedicated Perl script. The best model for nucleotide evolution was determined with ModelTest 3.7 [45] in combination with PAUP* (D. L. Swofford, 2003. PAUP*, Sinauer Associates, Sunderland, MA) as chosen by the Akaike Information Criterion (AIC). The evolutionary model selected was the general time reversal (GTR) model with a discontinuous gamma distribution (Γ) for modeling rate heterogeneity over sites and a proportion of invariant sites (I), *i.e.* GTR+Γ+I with four rate categories. The best fitting sequence substitution model for the protein data was determined with ProtTest 2.4 [46], and was found to be, according to the AIC, LG+Γ+I [47]. Also the second best model, JTT+Γ+I [48], was tested for phylogenetic tree construction.

### Phylogenetic Analysis

Bayesian inference of phylogeny was carried out with MrBayes 3.1.2 [49], [50] with default heating parameters (three heated Markov chain Monte Carlo chains and one cold) and priors. Two simultaneous and independent runs were carried out with sampling every 10 of 500 k generations until average standard deviation of split frequencies were below 0.01. Branch lengths and majority rule consensus tree topologies were calculated after discarding a burn-in of 100 k generations after which stationarity had been reached. For all calculations presented, the final potential scale reduction factor (PSRF) was below 1.004 for all parameters.

Phylogenetic trees were also generated with the Maximum Likelihood (ML) method employing PhyML 3.0 [51] with default parameters. The robustness of each clade was estimated by a nonparametric bootstrap analysis with 1000 replicates. Gamma distribution parameters, the proportion of invariable sites, branch lengths and GTR model parameters were all optimized by the ML algorithm from the data. ML phylogenetic trees were also inferred using RAxML 7.2.6 [52], but due to the similarities of the resulting trees only the PhyML results are shown here.

Dendroscope 2.7.4 [53] was used for visualization of phylogenetic trees. Signature sequence logos were generated by applying WebLogo [54]. Calculations were carried out on the University of Oslo Titan computer cluster, mainly through the freely available Bioportal (www.bioportal.uio.no). Protein structure illustrations were generated with PyMOL (W. L. DeLano, The PyMOL Molecular Graphics System, Version 1.3, Schrödinger, LLC). Ratios of nonsynonymous and synonymous substitution rates were calculated with the KaKs_Calculator [55].

## Results and Discussion

### The PKA Cα/Cβ Family is Highly Conserved in Chordates

Vertebrate homologs of human PKA Cα and Cβ were extracted from public databases, including Ensembl, UniProt, and database resources provided by the NCBI. New gene models were generated for several of the homologs by careful manual curation (See details in Materials and Methods S1). Homologs, in most cases full-length sequences, were found from 21 placental mammals, the marsupial species opossum (*Monodelphis domestica*) and wallaby (*Macropus eugenii*), chicken, zebra finch (*Taeniopygia guttata*), the Carolina anole lizard (*Anolis carolinensis*), and two and six species of frogs and bony fishes, respectively. In addition, full-length sequences were obtained for the non-vertebrate chordates amphioxus (*Branchiostoma floridae*) and the tunicates *Ciona intestinalis* and *Ciona savignyi*, the echinoderm sea urchin (*Strongylocentrotus purpuratus*), the two mollusks great pond snail (*Lymnaea stagnalis*) and California sea hare (*Aplysia californica*), the hemichordate *Saccoglossus kowalevskii*, the nematode *Caenorhabditis elegans*, the sponge *Amphimedon queenslandica,* as well as six arthropods including honey bee, fruit fly, a tick, and a crustacean. Partial sequences were obtained for orthologs from dogfish shark (*Squalus acanthias*), little skate (*Leucoraja erinacea*) and sea lamprey (*Petromyzon marinus*). In all cases both the nucleotide sequences and the corresponding protein sequences were stored. All sequences are listed in Materials and Methods S1.

We were unable to detect more than a single, reasonably close, PKA Cα/Cβ homolog in any of the invertebrate species that were examined. These includes the cephalochordate amphioxus, the urochordates *C. intestinalis*, *C. savignyi* and *Oikopleura dioica,* the echinoderm sea urchin, as well as arthropods, mollusks, a nematode and a sponge. Two homologs were found in most vertebrates, including sea lamprey, and the chondrichthyes *S. acanthias* and *L. erinacea,* while four homologs were detected in a number of bony fishes.

The main isoforms of human *PRKACA* and *PRKACB* comprises exons 2–10 and in addition one or more 5′ exons (Fig. 1A). The length of all exons 2–10 and the positions and intron phases of all nine introns are identical in the two human genes (Fig. 1B), clearly demonstrating that these genes arose due to a gene duplication. This structure, reflecting an extreme degree of conservation, is also conserved in all chordate homologs, including the invertebrate cephalochordate amphioxus and the urochordate tunicates and in addition in the echinoderm sea urchin, the hemichordate *S. kowalevskii*, and the crustacean *Daphnia pulex*: the number of exons, their lengths and the codon phases of all introns are identical. The exceptions, apart from the intronless homologs described below, includes the medaka gene encoding a protein with Ensembl identifier ENSORLP00000018527. This gene appears to have acquired a new 88-nucleotide intron (intron phase 0) in the middle of exon 9. In addition, the basal metazoan *A. queenslandica* has an additional intron of 334 nucleotides that splits the coding sequence corresponding to vertebrate exon 6.

The number of codons encoded by exons 2–10 in the *PRKACA*/*B* family of genes - corresponding to residues 16–350 in human proteins PKA Cα1/Cβ1 - is consequently also identical in all species listed above. We term this sequence segment Core_16–350_ (Fig. 1B) in order to distinguish it from the variable 5′ exons. The genomic data for the mollusks are not yet available in the public domain and the intron/exon structure is currently unknown, but in insects the protein coding segment of the *PRKACA/B* homologs are contained in a single exon. Nevertheless, the insect homologs also have the same number of residues for the segment corresponding to Core_16–350_. An MSA of all sequences with full-length Core_16–350_ is shown in Figure S1. Consequently, we find that nearly all our PKA Cα/Cβ homologs from Bilateria have the same length for the Core_16–350_, and with variable length N-termini corresponding to the multitude of isoforms. The only exceptions are a single-residue insertion after human PKA Cα1 Lys63, in the middle of exon 3, in both homologs from mollusks and a single-residue insertion after human PKA Cα1 Ala38 in a fast-evolving sequence from opossum (identifier ENSMODP00000015141). These insertions are in loop structures in PKA Cα1 (See *e.g.* [56] for the 3D structure) and are expected to be compatible with an unchanged overall 3D structure of the kinase.

Pairwise sequence identity between any of the bilaterian PKA Cα/Cβ homologs described above is always above 77% for the 335 residues of the Core_16–350_ at the amino acid level. Leaving out the fast-evolving sequences from marsupials and a single fast-evolving zebrafish sequence (Q7T374), the sequence identities are always above 80% and 87% within the chordates and vertebrates, respectively. This demonstrates very strong purifying selection and an exceptionally high degree of sequence conservation for this protein family.

### The PKA Cα/Cβ Gene Family Contains Several Putative Retroposons

In addition to the *PRKACA/B* homologs that have conserved exon/intron structure in chordates and several other deuterostomes, and the arthropod homologs, also with several exons, but with the protein coding segment contained in a single exon, a number of intronless *PRKACA/B* homologs are found in vertebrate genomes. Among these are human *PRKACG*, located on chromosome 9 between the genes *PIP5K1B* and *FXN*, but transcribed in the opposite direction. Intronless *PRKACG* has the same number of codons as the PKA Cα1-like transcript of *PRKACA* and is conserved in the great apes, in chimpanzee, gorilla, and orangutan and in the Old World monkeys rhesus macaque and hamadryas baboon (Sequences are listed in Materials and Methods S1). Genome browsing at the Ensembl resource also shows the synteny to be conserved in these species with *PIP5K1B* and *FXN* being transcribed in one direction and *PRKACG*, located between these two genes, in the opposite. Between *PIP5K1B* and *FXN* in the gibbon (*Nomascus leucogenys*) genome, a species more closely related to great apes than the Old World monkeys, there is no full-length *PRKACG* ortholog, but instead a putative *PRKACA/B* pseudogene with several frame shifting mutations. We find no evidence of *PRKACG* orthologs in prosimian primates such as greater galago (*Otolemur garnettii*), tarsier (*Tarsius syrichta*), or gray mouse lemur (*Microcebus murinus*), although the last two of these have genomes that are still fragmented and with undisclosed synteny around the genes *PIP5K1B* and *FXN*. In the common marmoset (*Callithrix jacchus*) genome, there is a fragment, most likely not protein-coding, of *PRKACG* between *PIP5K1B* and *FXN*, corresponding to PKA Cα1 residues 21–350. Finally, in the mouse and dog genomes, *PIP5K1B* and *FXN* are neighboring genes being transcribed in the same direction, but without any sign of a *PRKACA/B* homolog in this region.

These findings strongly support the previous suggestion that *PRKACG* is a retroposon due to a PKA Cα1-type transcript [35] that has been inserted between *PIP5K1B* and FXN in a common ancestor of great apes and Old and New World monkeys. The putative *PRKACG* transcripts could potentially give rise to functional kinases in all great apes and in the Old World monkeys, but not in gibbons and the New World monkey marmoset where there are mutations disrupting the reading frame in the *PRKACG* retroposon. In the marmoset genome, there is in addition to the putative *PRKACG* pseudogene on chromosome 1, a second retroposon (Ensembl identifier ENSCJAP00000040924) related to PKA Cα1 on chromosome 2. This gene was not found in other primates.

In addition to the PKA Cα1-like retroposons in primates, we found intronless *PRKACA* homologs in the two sequenced genomes of marsupials, the wallaby kangaroo (*M. eugenii*) and the Brazilian opossum (*M. domestica*) (See Materials and Methods S1). Also these putative retroposons appear to be derived from a PKA Cα1-type transcript, but are otherwise unrelated to primate *PRKACG*.

### Elucidating the Phylogeny of the PKA Cα/Cβ Family is Nontrivial Due to High Sequence Conservation

**Figure 2:**
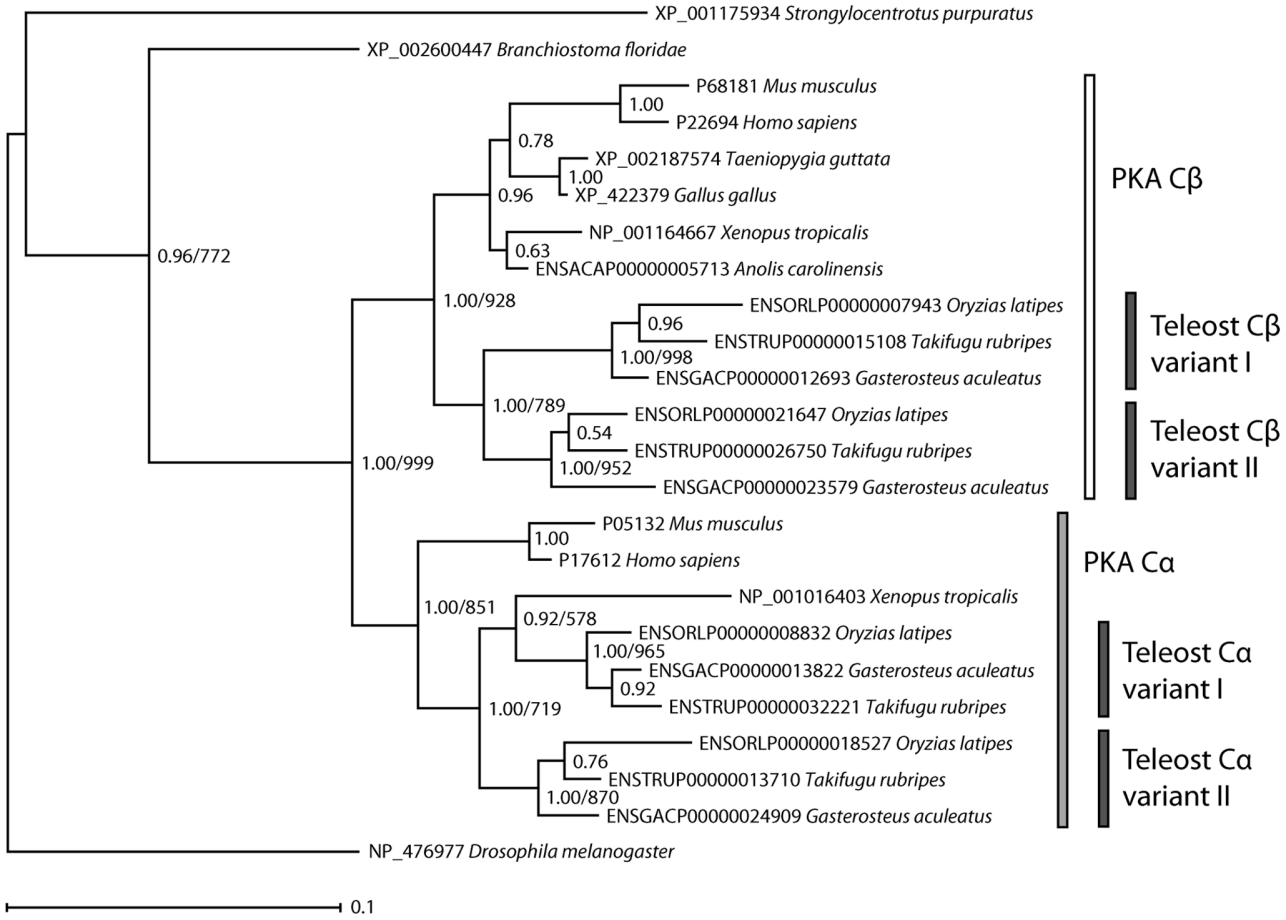
Phylogenetic relationships among the PKA catalytic subunit homologs in chordates. The Cα and Cβ paralogs are a result of a gene duplication in a common ancestor of vertebrates. Subsequent duplications of Cα and Cβ in a teleost fish ancestor have resulted in four PKA catalytic subunits in these organisms. The Bayesian inference tree is based on the nucleotide sequences (codon positions 1 and 2 only, GTR+Γ+I model) of exons 2 to 10 which corresponds to a multiple sequence alignment with no gaps. The phylogram is shown with estimated branch lengths proportional to the number of substitutions at each site, as indicated by the scale bar. The arthropod fruit fly (*D. melanogaster*) and the echinoderm sea urchin (*S. purpuratus*) have been set as outgroups. Bayesian posterior probabilities are shown for each node. The topology of a maximum likelihood (ML) tree generated with the same data set and model was identical to the Bayesian inference tree. ML bootstrap values are shown for selected nodes (1000 replications). The sequences of human and mouse PKA Cα and Cβ and the homologs from amphioxus (*B. floridae*), zebra finch (*T. guttata*), chicken (*G. gallus*), the frog *X. tropicalis*, the lizard *A. carolinensis*, medaka (*O. latipes*), the pufferfish *T. rubripes*, and stickleback (*G. aculeatus*) are described in Materials and Methods S1. The *X. tropicalis* Cα and *A. carolinensis* Cβ are incorrectly placed (See discussion and Fig. 3).

**Figure 3:**
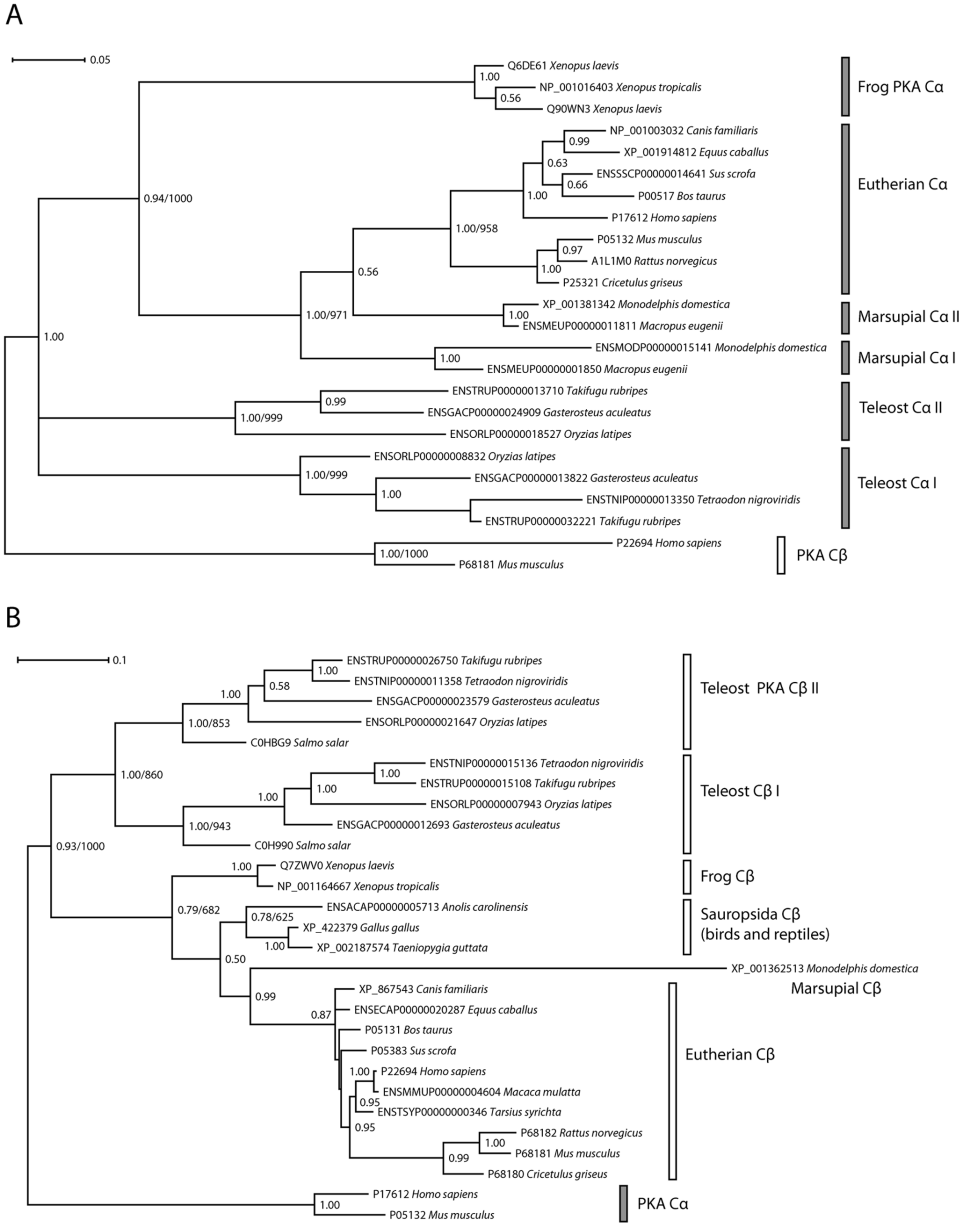
The Bayesian inference trees for vertebrate PKA Cα and Cβ both closely reflects the evolutionary relationships among these organisms. **A** Phylogenetic analysis of Cα orthologs resulted in a tree that was rooted with human and mouse Cβ as outgroups. The tree was based on the nucleotide sequences of exons 2 to 8 (all codon positions, GTR+Γ+I model). **B** Phylogenetic analysis of Cβ orthologs was performed employing nucleotide sequence data (all codon positions, exons 2 to 10, GTR+Γ+I model). The resulting tree was rooted with human and mouse Cα as outgroups. In both trees, branch lengths are shown as substitutions per site, with scale indicated by the scale bars. Bayesian posterior probabilities are given for each node and ML bootstrap values (1000 replications) are shown for selected nodes where the clades are identical in the Bayesian and ML analysis. In addition to organisms found in Fig. 2, representative sequences from the following species were included: eutherian mammals rhesus macaque (*M. mulatta*), tarsier (*T. syrichta*), dog (*C. familiaris*), horse (*E. caballus*), pig (*S. scrofa*), cow (*B. taurus*), rat (*R. norvegicus*), and hamster (*C. griseus*), marsupial mammals wallaby (*M. eugenii*) and opossum (*M. domestica*), the frog *X. laevis*, the pufferfish *T. nigroviridis* and Atlantic salmon (*S. salar*). See Materials and Methods S1 for the sequence data.

The distribution of PKA Cα/Cβ homologs in Bilateria, as well as phylogenetic trees generated with unsophisticated hierarchical clustering methods (results not shown), suggest that this gene family has expanded through repeated gene duplication events in vertebrates. However, despite numerous attempts, state-of-the-art probabilistic methods, both Bayesian inference and maximum likelihood (ML) methods, were not able to generate a statistically strongly supported phylogenetic tree for the full PKA Cα/Cβ family from the complete data set. After careful analysis of the data (*vide infra*), we were nevertheless able to derive reliable phylogenies for this gene family by dividing the data into subsets. The final phylogenetic trees were generated as follows: an MSA was generated from the nucleotide sequences corresponding to the Core_16–350_ segment for selected vertebrates, amphioxus, sea urchin and fruit fly. After removal of all nucleotides at codon position 3, the dataset was employed to generate Bayesian inference and ML trees, with identical topology, for the PKA Cα/Cβ homologs with good Bayesian posterior probabilities and bootstrap support for the major nodes. The phylogram was rooted with the sea urchin and fruit fly as outgroups (Fig. 2). Bayesian inference methods were also used to generate a phylogenetic tree of 22 vertebrate PKA Cα orthologs employing human and mouse PKA Cβ as outgroups (Fig. 3A). Similarly, a tree with 26 PKA Cβ orthologs was generated with human and mouse PKA Cα as outgroups (Fig. 3B). These trees, and trees from the corresponding ML analysis, are based on MSAs for the nucleotide sequences with all three codon positions included.

Neither Bayesian inference nor ML methods, generally accepted to be the most accurate [57], [58], were able to generate a reliable phylogeny for the PKA Cα/Cβ family from the full data set. This was not due to unreliable MSAs as only three of the sequences in the original data set each had single codon insertions (*vide supra*) and manual removal of these was trivial. The problem, however, appears to be a combination of weak phylogenetic signals and problematic nonphylogenetic signals in the data. Due to the very high level of sequence conservation, the protein data set contains few phylogenetically informative sites, *i.e.* amino acid sites that favor one phylogenetic tree topology over others. As an example, for the 22 chordate taxa used to generate the tree in Fig. 2, the corresponding amino acid data set has 335 columns/sites. Of these, only 58 sites are phylogenetically informative, while 255 are fully conserved and identical for all taxa and the remaining 22 are autapomorphic. Unsurprisingly, protein data phylogenetic trees generated with two recommended substitution models (LG/JTT+Γ+I) and well-tested ML programs (PhyML/RAxML) were overall fairly similar, but with very poor bootstrap support. The trees generated with various subsets of the available taxa were incongruent and also in several cases inconsistent with the known evolutionary relationship between the various chordate species.

In order to secure a stronger phylogenetic signal [59], the nucleotide data set was employed for deriving the phylogenetic relations. For the 22 chordate taxa in Fig. 2, there are 77, 33, and 312 phylogenetically informative sites (out of 335 sites in total) for codon position 1, 2, and 3, respectively. The low number of informative sites at codon position 1 and 2 reflects the high degree of conservation at the amino acid level, while the high number at codon position 3 (93%) reflects the large time-span since the common ancestor of these genes. For all tested nucleotide data sets, the GTR+Γ+I model was predicted to be superior. While using the nucleotide sequences for deriving the phylogenetic relationships ensures a stronger phylogenetic signal, in particular the data from codon position 3, due to the degeneracy of the genetic code, may be severely mutationally saturated due to reversions and convergences that erase the true phylogenetic signal. This will especially be problematic for inferring ancient phylogenies [59], where long branch attraction (LBA), *i.e.* a tendency for grouping of lineages with long branches irrespective of their true relationships, may lead to misleading phylogenies [60]. In particular, fast-evolving genes may artificially occur too deeply in the tree due to LBA towards the outgroups.

As for the protein data set, trees generated with subsets of the available taxa were highly incongruent and in several cases with pronounced LBA, clearly demonstrating a strong nonphylogenetic signal. In order to secure minimal nonphylogenetic signals and reduce LBA, codon position 3 sites were removed from the data set used to generate the phylogeny in Fig. 2. In addition, fast-evolving taxa such as the tunicate [61], marsupial PKA Cα/Cβ homologs and the primate PKA Cγ homologs were left out of the data set. Fig. 2 is expected to describe the ancient evolution of the PKA Cα/Cβ family in chordates correctly, with the gene duplications in the common ancestor of vertebrates as well as in teleost fishes supported by high Bayesian posterior probabilities and strong bootstrap support for the ML analysis.

In order to better describe the evolution of the two PKA Cα and PKA Cβ paralogs in vertebrates separately, the trees in Fig. 3 were generated. Reliable phylogenies could not be generated from a data set containing codon positions 1 and 2 only, as in Fig. 2, most likely due to weak phylogenetic signals in the data. However, for the relatively recent phylogenetic relations in Fig. 3, the saturation at codon position 3 is expected to be less severe and all three codon positions were included in the data set for analysis. In Fig. 2, there are two errors due to nonphylogenetic signal. These are the placement of frog PKA Cα in a clade together with teleost fish and the erroneous lizard/frog PKA Cβ clade. Both these errors are corrected in Fig. 3 upon inclusion of codon position 3 data. The most ancient splittings, however, are unreliable in Fig. 3, in particular the description of the branching between the tetrapod and teleost fish PKA Cα homologs as a multifurcation (Bayesian analysis) or two bipartitions with extremely poor bootstrap support (ML analysis, not shown) in Fig. 3A.

In conclusion, while the phylogenies of the PKA Cα and PKA Cβ subfamilies, especially within the tetrapods, appears to be correctly inferred from the full nucleotide dataset (Fig. 3), the ancient evolution of the PKA Cα/Cβ family in chordates is correctly described with cladistic methods only after removal of codon position 3 data (Fig. 2). The protein data set contains limited phylogenetic information and does not give reliable phylogenies in chordates. No analysis based on partitioned data, for example according to codon position, was attempted, as this is likely to lead to overparametrization.

### Phylogenetic Inferences for the PKA Cα/Cβ Family in the Chordate Lineage

The well-resolved phylogenetic tree in Fig. 2 strongly suggests the following sequence of events during the evolution of the PKA Cα/Cβ gene family: a single PKA Cα/Cβ-like gene in the common ancestor of chordates, arthropods and echinoderms was duplicated in a common ancestor of vertebrates, which lead to the two paralogous genes corresponding to PKA Cα and Cβ. These two genes were again duplicated in a common ancestor of teleost fishes, leading to paralogs that we suggest are denoted PKA Cα-I, Cα-II, Cβ-I, and Cβ-II.

The data indicates that the first PKA Cα/Cβ gene duplication, resulting in PKA Cα and PKA Cβ, took place after the divergence of the urochordate and cephalochordate lineages. Currently there are only fragments of PKA Cα/Cβ homologs available in public databases for the chondrichthyes (dogfish shark and little skate) and the cyclostome sea lamprey (Table S1), but the PKA Cα/Cβ homologs also in these organisms appear to occur in pairs. Unfortunately, the phylogenetic signal in the data is too weak to classify these sequences as PKA Cα or PKA Cβ, and to exclude the possibility that these paralogous gene pairs are results of independent gene duplications, but the most parsimonious explanation for this distribution of PKA Cα/Cβ homologs is that a single PKA Cα/Cβ gene duplication occurred before the divergence of the jawless fish lineage and the subsequent divergence of sharks and skates. Interestingly, this timing of the PKA Cα/Cβ gene duplication coincides with the two rounds (2R) of whole genome duplication (2R hypothesis) that took place after the emergence of the invertebrate chordates and before the radiation of jawed vertebrates [62]–[64]. The PKA Cα and PKA Cβ gene split is thus likely to have occurred in the Cambrian, roughly 500 Mya [65], [66]. Canaves and Taylor [38] found that the gene duplications of the PKA regulatory subunits resulting in the paralogs RIα and RIβ as well as RIIα and RIIβ also occurred in the chordate lineage, suggesting that the gene duplications of the R and C subunits might have taken place simultaneously.

The secondary duplications of PKA Cα and PKA Cβ (Figs. 2 and 3) appear to be unique to teleost fishes, and might have coincided with the teleost whole genome duplication that took place 226–316 Mya [67], [68]. Finally, the presence of two *X. laevis* PKA Cα paralogs and a single *X. tropicalis* PKA Cα (Fig. 3A) in the amphibian genomes is consistent with the recent whole genome duplication event in the common ancestor of the *X. laevis* group not found in *X. tropicalis* [69], [70].

As expected, both the subtrees for PKA Cα (Fig. 3A) and PKA Cβ (Fig. 3B) have eutherian clades with the marsupial orthologs as sister clades, and with Sauropsida (birds and reptiles, Fig. 3B only) and frogs appearing as sister clades deeper into the phylogenetic tree. We were unable to find the PKA Cα gene in any of the released genomes of Sauropsida, and this clade is consequently missing in Fig. 3A. However, a single EST (expressed sequence tag) sequence from a chicken testis library (GenBank identifier CN229123 [71]) appears to confirm the presence of PKA Cα also in birds. Several vertebrate species are missing either PKA Cα or PKA Cβ in the current genomic data sets, but this is most likely due to low sequence coverage in unfinished genomes. We find no evidence for extensive PKA Cα or Cβ gene loss in any of the main vertebrate groups.

The marsupial *M. domestica* PKA Cβ appears to be particularly fast-evolving (Fig. 3B). The *M. eugenii* PKA Cβ ortholog is present in the genome, but the full sequence is currently unknown. Both marsupial genomes [72], [73] have two PKA Cα paralogs. Marsupial PKA Cα-I has the same exon/intron structure as PKA Cα in all other mammals. Marsupial PKA Cα-II, however, is intronless and a putative PKA Cα retroposon (Fig. 3A). The full-length sequences are not at present available for all the marsupial PKA Cα homologs, but interestingly, intronless PKA Cα-II appears to be significantly more conserved than the ancestral variant PKA Cα-I. The 5′ segment of the two marsupial PKA Cα-II orthologs (only the 232 5′ codons are available in the sequence for *M. eugenii*) have 17 synonymous and no non-synonymous mutations, suggesting strong purifying selection. For PKA Cα-I, the 294 codons corresponding to exons 2–9 (exons 1 and 10 are missing in the current *M. eugenii* genomic sequence) have 70 mutations, including a 3 nucleotide insertion in opossum PKA Cα-I, and 21 of these are non-synonymous. These data, although limited, strongly suggests that the retroposon PKA Cα-II have become functional in marsupials and that the purifying selection acting upon PKA Cα-I and PKA Cβ has become less stringent.

### Vertebrate PKA Cα and Cβ Mainly Differs in the C-tail and in Subdomains I and II

**Figure 4:**
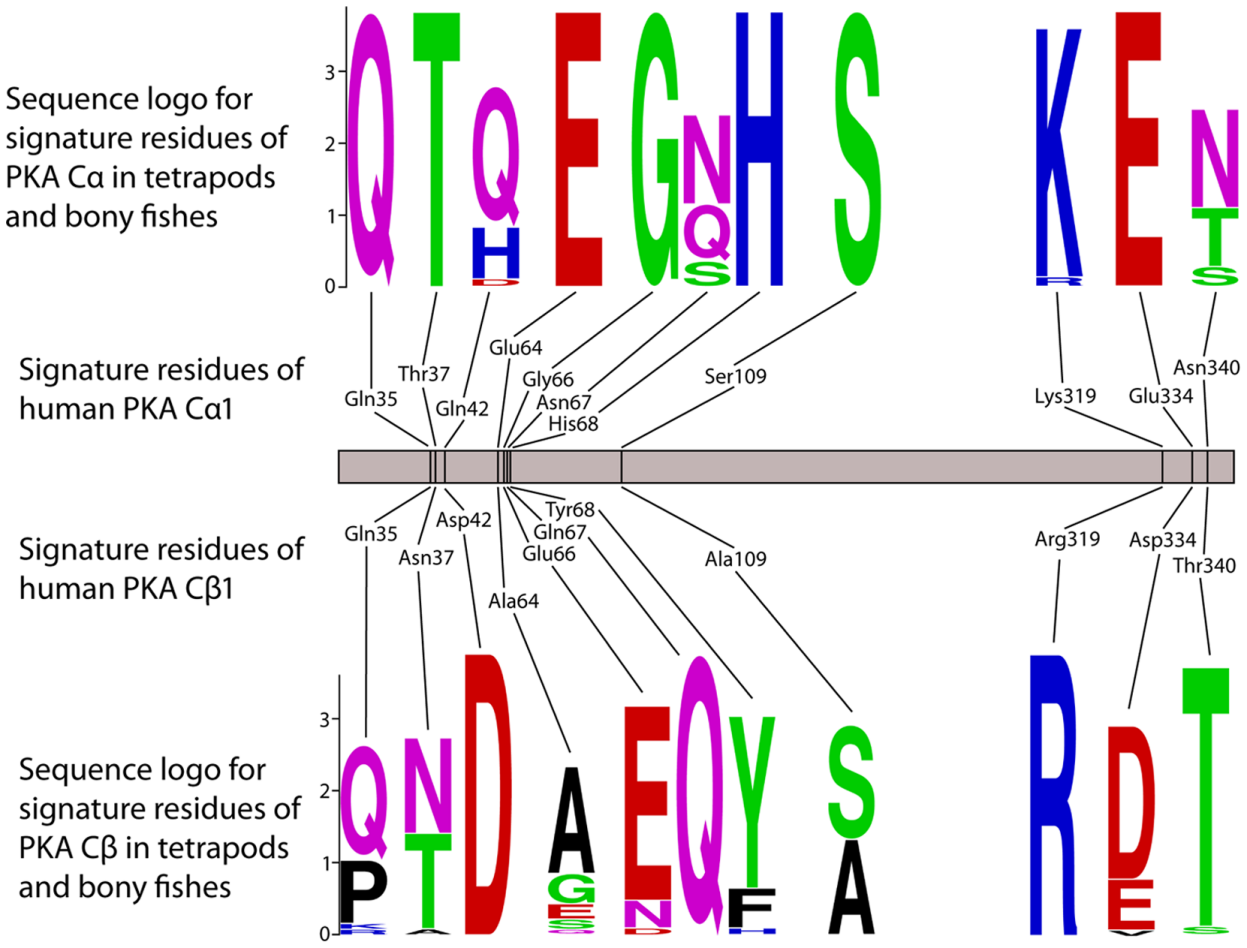
The identity of eleven amino acids in the protein chain may define the Cα and Cβ branches of PKA catalytic subunits. Our full set of PKA catalytic subunits (Materials and Methods S1) from bony fishes and tetrapods, comprising 27 Cα and 33 Cβ, was employed to identify eleven amino acid positions that together may be used to classify a PKA catalytic subunit as belonging to one of the two branches. The sequence logos define the PKA Cα and Cβ clades within the *Teleostomi*, which includes the familiar classes of bony fishes, birds, mammals, reptiles, and amphibians. We find invariable Gln35, Thr37, Glu64, Gly66, His68, Ser109 and Glu334 in Cα and invariable Asp42, Gln67, and Arg319 in Cβ (Cα1/Cβ1 numbering). The residues in the corresponding positions in human Cα1 and Cβ1 are also shown.

In order to elucidate the potential differences between the PKA Cα and PKA Cβ protein subfamilies, an MSA of all available vertebrate homologs was generated. In this set of 27 PKA Cα and 33 PKA Cβ, there are within the 335 sites/columns of the Core_16–350_ only 62 sites (19%), that are phylogenetically informative while 235 sites are fully conserved in all taxa, again reflecting the very high degree of purifying selection. A manual inspection of the MSA showed that eleven sites/columns could tentatively be used to discriminate between the two PKA subfamilies. Sequence logos for these eleven sites are shown in Fig. 4. Human PKA Cα1 residues Gln35, Thr37, Glu64, Gly66, His68, Ser109, and Glu334 are fully conserved in all vertebrate PKA Cα, while at the corresponding sites in PKA Cβ the sequence conservation is less stringent. Similarly, human PKA Cβ1 residues Asp42, Gln67, Arg319 are fully conserved in vertebrate PKA Cβ (Fig. 4). The single site where there is no overlap between amino acid use in the two subfamilies is at residues 66 where PKA Cα and Cβ have Gly and Glu/Asn/Asp, respectively.

**Figure 5:**
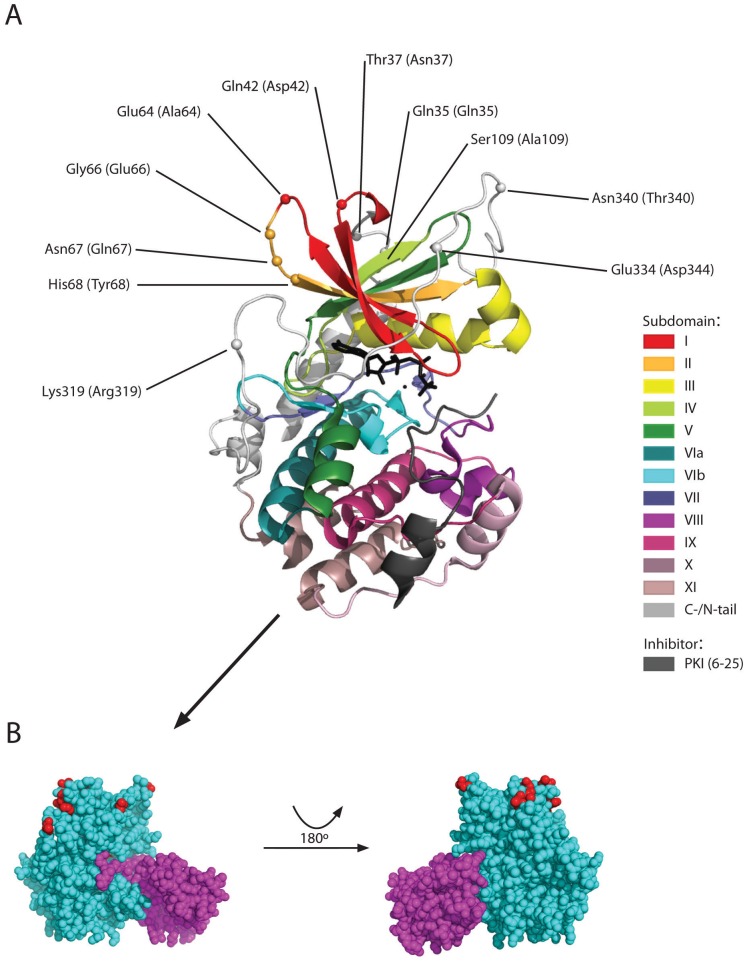
Signature residues defining PKA Cα and Cβ do not interact with ATP, peptide inhibitor PKIα or the kinase regulatory subunit. **A** The tentative signature residues of PKA Cα and Cβ (Fig. 4) are highlighted in a structural model of PKA Cα1 in complex with a truncated PKIα (residues 6–25). Signature amino acids in human Cα1 and Cβ1 are shown without and within parenthesis, respectively. The conserved kinase core has been divided into subdomains (represented in different colors) [87] as defined by Hanks and Hunter [88]. ATP is rendered as sticks (black) and two divalent cations as black spheres. The model is based on the experimental structure of Thompson *et al*. [56] (PDB identifier 3FJQ). **B** Residues in Cα1 (cyan) interacting with regulatory subunit RIα (purple, residues 92–245 of bovine RIα only) are mainly restricted to the large lobe and do not overlap with any of the Cα signature residues (red). The complex is shown with the same orientation of Cα1 as in panel A (left) and rotated 180° (right). The model is based on the experimental structure of Kim *et al.* [5] (PDB identifier 3FHI).

The residues that correspond to the eleven sites that tentatively discriminates between the subfamilies PKA Cα and Cβ are all exposed at the protein surface, in or close to loop structures, in subdomains I and II and in the C-tail (Fig. 5). None of these residues are located close to the kinase active site and their identity is not likely to affect PKA kinase activity. Likewise, they are not located near the protein surface segments that are known to interact with the PKA regulatory subunits (Fig. 5B), and these residues should not be important for R subunit interactions. Several of the eleven residues, especially residues 64, 319, 334 and 340, are protruding their side chains into the solvent and might be targets for PKA Cα- or PKA Cβ-specific post-translational modifications and/or protein-protein interactions. Particularly, Glu64 is conserved in all vertebrate PKA Cα, while residue 64 is variable in 33 Cβ, being Ala in all mammals and Glu in only three fish homologs. In PKA Cβ Glu66 is absolutely conserved, except in five of the fish homologs, while this residue is conserved as Gly in Cα.

### Strong Purifying Selection is Lost in PKA Cγ

A comparison of PKA Cα1 from human and the galago, a prosimian primate, shows that there are 59 and 4 synonymous (silent) and non-synonymous (amino acid changing) mutations, respectively. Between human and macaque PKA Cγ there are slightly fewer, 44, mutations, but from these 31 amino acid changes, indicating that the strong purifying selection in the PKA Cα lineage is lost in PKA Cγ. A powerful tool for evaluating the evolution of protein coding sequences, is calculating the ratio of the non-synonymous (*K*a) and synonymous (*K*s) substitution rates, where *K*s is the number of synonymous substitutions per synonymous site and *K*a is the number of non-synonymous substitutions per non-synonymous site [74]. A *K*a/*K*s <1 indicates purifying (negative) selection, *K*a/*K*s >1 is a sign of positive selection, while *K*a/*K*s ∼ 1 indicates neutral evolution of the protein. Table 1 shows *K*a/*K*s for comparisons of five representative tetrapod PKA Cα homologs and five primate PKA Cγ homologs. Due to missing sequences in the databases it was not possible to compare the same species for Cα and Cγ. For the PKA Cα sequences, the average *K*a/*K*s is 0.011. PKA Cα sequences were also compared for other placental mammals and *K*a/*K*s were found to be in the range 0.004–0.023. *K*a/*K*s for a comparison of human PKA Cα and Cβ, and of the human and mouse PKA Cβ orthologs, were 0.0076 and 0.0198, respectively. These data for the PKA Cα/Cβ homologs in placental mammals again confirms the very strong purifying selection acting upon these kinases.

**Table 1:** Ratio of amino acid replacing (*K*a) and silent (*K*s) mutation rates for all pairwise comparisons of five tetrapod PKA Cα homologs and five primate PKA Cγ homologs.

	*K*a/*K*s^a^			
	*H. sapiens* Cα	*O. garnettii* Cα	*M. musculus* Cα	*B. taurus* Cα
*O. garnettii* Cα	0.0159 (0.0158)			
*M. musculus* Cα	0.0141 (0.0141)	0.0230 (0.0226)		
*B. taurus* Cα	0.0064 (0.0063)	0.0116 (0.0111)	0.0156 (0.0158)	
*X. tropicalis* Cα	0.0065 (0.0060)	0.0055 (0.0056)	0.0065 (0.0064)	0.0059 (0.0060)
	***K*** **a/** ***K*** **s** b			
	***H. sapiens*** ** Cγ**	***P. troglodytes*** ** Cγ**	***P. pygmaeus*** ** Cγ**	***G. gorilla*** ** Cγ**
*P. troglodytes* Cγ	0.135 (0.127)			
*P. pygmaeus* Cγ	0.290 (0.324)	0.175 (0.194)		
*G. gorilla* Cγ	1.360 (1.207)	0.162 (0.148)	0.295 (0.319)	
*M. mulatta* Cγ	0.697 (0.678)	0.419 (0.406)	0.393 (0.393)	0.621 (0.603)

a *K*a/*K*s ratios for pairwise comparisons of PKA Cα from human, a prosimian primate (*O. garnettii)*, mouse (*M. musculus*), cattle (*B. taurus*) and a frog (*X. tropicalis*) calculated according to the model of Goldman and Yang [89] and the model averaging method of Zhang *et al.* (in parenthesis) [90] for the Core_16–350_ sequence segment.

b *K*a/*K*s ratios for pairwise comparisons of PKA Cγ from human, chimpanzee (*P. troglodytes*), orangutan (*P. pygmaeus*), gorilla (*G. gorilla*) and rhesus macaque (*M. mulatta*) calculated as described above.

The *K*a/*K*s values from the comparison of the primate PKA Cγ sequences in Table 1 are in the range 0.14–1.36. Due to the fairly close evolutionary relationships between these species, the number of mutations in *PRKACG* transcripts between these species is rather low, for example 9, 31 and 46 between human and chimpanzee, orangutan, and macaque, respectively. Consequently, the *K*a/*K*s values are not expected to be highly reliable, but the average value of 0.45 clearly suggests that the strong purifying selection in the PKA Cα lineage is lost in PKA Cγ. This finding that mutations in PKA Cγ appear to be neutral, combined with the loss of a functional Cγ in gibbons and marmoset (*vide supra*), suggest that there are no evolutionary constraints on maintaining a functional PKA Cγ protein in higher primates. However, this analysis does not exclude the possibility that the PKA Cγ transcript has an important function in humans, great apes and other Simiiformes, for example in regulation of PKA Cα/Cβ transcript processing [75].

### Conclusion

We have shown that the PKA Cα and Cβ catalytic subunits found in chordates and other animal species builds a phylogenetic clade of kinases with a very high degree of conservation at the protein level. In the core segment corresponding to exons 2–10 of vertebrate Cα1/Cβ1 the synonymous mutation rate is approximately two orders of magnitude larger than the amino acid changing mutation rate. All the main residues and sequence segments previously shown to be important for human Cα/Cβ function (Fig. 1C), including the phosphorylation sites, the ATP and Mg^2+^ interacting residues and the DFG and P+1 motifs, are basically fully conserved in all homologs in chordates, insects and other animal sequences investigated in the current study. The few residues that differ in Cα and Cβ (Fig. 4) should be investigated in order to elucidate possible functional differences between the two paralogs. Finally, the Cα1-derived expressed retroposon Cγ found in higher primates appears to be evolving neutrally and appears to have no function as a mature protein.

## Supporting Information

Figure S1 **Multiple sequence alignment of segment corresponding to exons 2–10 of human PKA Cα1 (** ***i.e.*** **residues 16–350).** All sequences are described in Materials and Methods S1. Residue numbering of human PKA Cα1 (identifier P17612) is shown above the sequence. Sequences belonging to the Cα, Cγ, and Cβ clades are marked at the right by a green, blue, and red bar, respectively. The figure was prepared with Jalview [44].
(PDF)

Table S1 **Sequence data for PKA catalytic subunit homologs from chordates collected and manipulated as described in Materials and Methods S1.**
(PDF)

Materials and Methods S1 **Details on sequence data collection.**
(PDF)
