# Supplementary material for: Evolutionary Paths of the cAMP-Dependent Protein Kinase (PKA) Catalytic Subunits
Source: PLoS One. 2013 Apr 12;8(4):e60935. doi: 10.1371/journal.pone.0060935 (PMC3625193; doi:10.1371/journal.pone.0060935)
Supplement: Figure S1 — Multiple sequence alignment of segment corresponding to exons 2–10 of human PKA Cα1 ( i.e. residues 16–350). All sequences are described in Materials and Methods S1. Residue numbering of human PKA Cα1 (identifier P17612) is shown above the sequence. Sequences belonging to the Cα, Cγ, and Cβ clades are marked at the right by a green, blue, and red bar, respectively. The figure was prepared with Jalview [44]. (PDF) [file pone.0060935.s001.pdf]

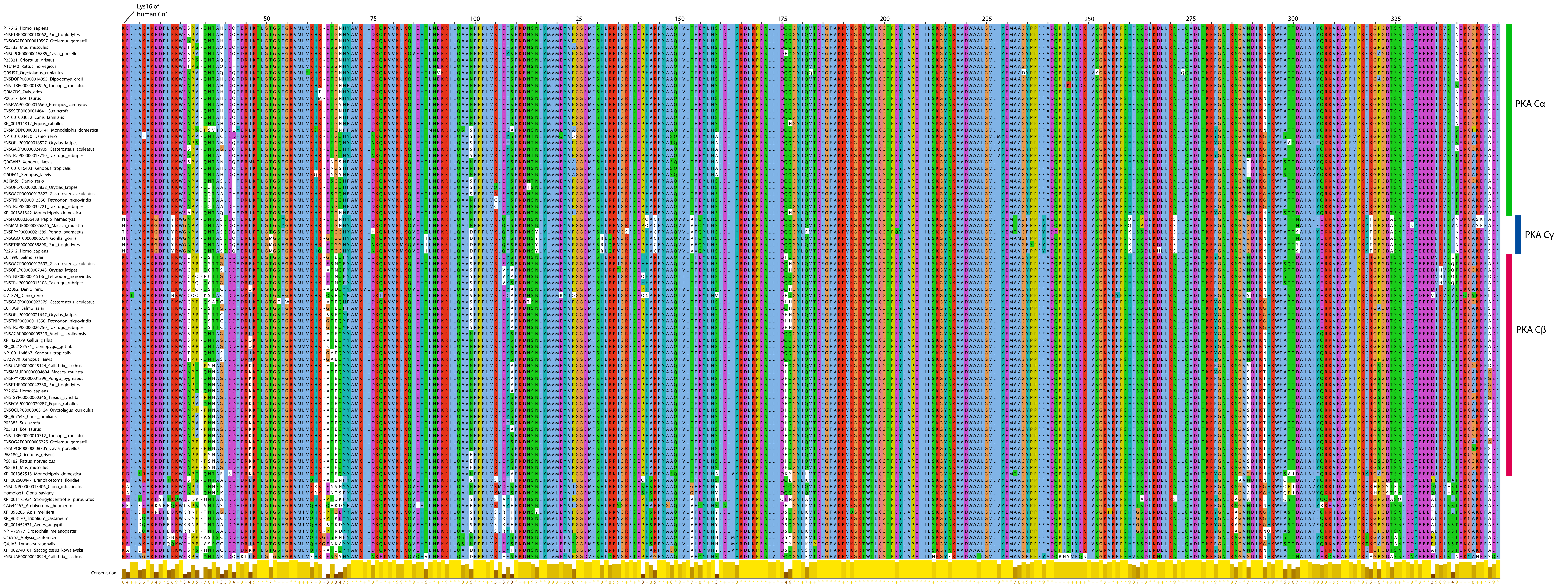


**FIGURE S1.** Multiple sequence alignment of segment corresponding to exons 2-10 of human PKA Cα1 (*i.e.* residues 16-350). All sequences are described in Materials and Methods S1. Residue numbering of human PKA Cα1 (identifier P17612) is shown above the sequence. Sequences belonging to the Cα, Cγ, and Cβ clades are marked at the right by a green, blue, and red bar, respectively. The figure was prepared with Jalview (http://www.jalview.org).
