# Supplementary material for: Evolutionary Paths of the cAMP-Dependent Protein Kinase (PKA) Catalytic Subunits
Source: PLoS One. 2013 Apr 12;8(4):e60935. doi: 10.1371/journal.pone.0060935 (PMC3625193; doi:10.1371/journal.pone.0060935)
Supplement: Table S1 — Sequence data for PKA catalytic subunit homologs from chordates collected and manipulated as described in Materials and Methods S1. (PDF) [file pone.0060935.s002.pdf]

TABLE S1. Sequence data for PKA catalytic subunit homologs from chordates collected and manipulated as described in Materials and Methods S1.

Species	PKA catalytic subunit homologs in FASTA format
P. troglodytes	>ENSPTRP00000018062_Pan_troglodytes (a)<br>MASNSSDVKEFLAKAKEDFLKKWESPAQNTAHLDQFERIKTLGTGSFGRVMLVKHKETGNHYAMKILDKQKV<br>VKLKQIEHTLNEKRILQAVNFPFLVKLEFSFKDNSNLYMVMEYVPGGEMFSHLRRIGRFSEPHARFYAAQIV<br>LTFEYLHSLDLIYRDLKPENLLIDQQGYIQVTDFGFAKRVKGRTWTLCGTPEYLAPEIILSKGYNKAVDWWA<br>LGVLIYEMAAGYPPFFADQPIQIYEKIVSGKVRFPSHFSSDLKDLLRNLLQVDLTKRFGNLKNGVNDIKNHK<br>WFATTDWIAIYQRKVEAPFIPKFKGPGDTSNFDDYEEEEIRVSINEKCGKEFSEF<br><br>>ENSPTRP00000042330_Pan_troglodytes (b)<br>MAAYREPPCNQYTGTTTALQKLEGFASRLFHRHSKGTAHDQKTALENDSLHFSEHTALWDRSMKEFLAKAKE<br>DFLKKWENPTQNNAGLEDFERKKTLGTGSFGRVMLVKHKATEQYYAMKILDKQKVVKLKQIEHTLNEKRILQ<br>AVNFPFLVRLEYAFKDNSNLYMVMEYVPGGEMFSHLRRIGRFSEPHARFYAAQIVLTFEYLHSLDLIYRDLK<br>PENLLIDHQGYIQVTDFGFAKRVKGRTWTLCGTPEYLAPEIILSKGYNKAVDWWALGVLIYEMAAGYPPFFA<br>DQPIQIYEKIVSGKVRFPSHFSSDLKDLLRNLLQVDLTKRFGNLKNGVSDIKTHKWFATTDWIAIYQRKVEA<br>PFIPKFRGSGDTSNFDDYEEEDIRVSITEKCAKEFGEF<br><br>>ENSPTRP00000035898_Pan_troglodytes (g)<br>MGNAPAKKDTEQEESVNEFLAKARGDFLYRWGNPAQNTASSDQFERLRTLGMGSFGRVMLVRHRETGGHYAM<br>KILNKQKVVKMKQVEHILNEKRILQAIDFPFLVKLQFSFKDNSYLYLVMEYVPGGEMFSRLQRVGRFSEPHA<br>CFYAAQVVLAFQYLHSLDLIHRDLKPENLLIDQQGYLQVTDFGFAKRVKGRTWTLCGTPEYLAPEIILSKGY<br>NKAVDWWALGVLIYEMAVGFSPFYADQPIQIYEKIVSGRVRFPSKLSSDLKDLLRSLLQVDLTKRFGNLRNG<br>VGDIKNHKWFATTSWIAIYEKKVEAPFIPKYTGPGDASNFDDYEEEELRISINEKCAKEFSEF
G. gorilla	>ENSGGOT00000006754_Gorilla_gorilla_translated (g)<br>MGNAPTKKDTEQEESMNEFLAKARGDFLYRWGNPAQNTASSDQFERLRTLGMGSFGRVMLVRHRGTGGHYAM<br>KILNKQKVVKMKQVEHILNEKRILQAIDFPFLVKLQFSFKDNSYLYLVMEYVPGGEMFSRLQRVGRFSEPHA<br>CFYAAQVVLAFQYLHSLDLIHRDLKPENLLIDQQGYLQVTDFGFAKRVKGRTWTLCGTPEYLAPEIILSKGY<br>NKAVDWWALGVLIYEMAMGFPPFYADQPIQIYEKIVSGRVRFPSKLSSDLKDLLRSLLQVDLTKRFGNLRNG<br>VGDIKNHKWFATTNWIAIYEKKVEAPFIPKYTGPGDASNFDDYEEEELRISINEKCAKEFSEF
P. pygmaeus abelii	>ENSPPYP00000001399_Pongo_abelii (b)<br>MAAYREPPCNQYTGTSTALQKLEGFASRLFHRHSKGTAHDQKTALQNDSLHFSEHTALWDRSMKEFLAKAKE<br>DFLKKWENPTQNNAGLEDFERKKTLGTGSFGRVMLVKHKATEQYYAMKILDKQKVVKLKQIEHTLNEKRILQ<br>AVNFPFLVRLEYAFKDNSNLYMVMEYVPGGEMFSHLRRIGRFSEPHARFYAAQIVLTFEYLHSLDLIYRDLK<br>PENLLIDHQGYIQVTDFGFAKRVKGRTWTLCGTPEYLAPEIILSKGYNKAVDWWALGVLIYEMAAGYPPFFA<br>DQPIQIYEKIVSGKVRFPSHFSSDLKDLLRNLLQVDLTKRFGNLKNGVSDIKTHKWFATTDWIAIYQRKVEA<br>PFIPKFRGSGDTSNFDDYEEEDIRVSITEKCAKEFGEF<br><br>>ENSPPYP00000021585_Pongo_abelii (g)<br>MGNAPAKKDTEQEKSATEFLVKARGDFLYRWGNPAQNTASSDQFERLRTLGTGSFGRVMLVRHRETGGHYAM<br>KILHKQKLVKLKQVEHTLNEKRILQAIDFPFLVKLQFSFKDNSYLYLVMEYVPGGEMFSHLRRVGSFSEPHA<br>CFYAAQVVLAFQYLHSLDLIHRDLKPENLLIDQQGYLRVTDFGFAKRVKGRTWTLCGTPQYLAPEIILSKGY<br>NKAVDWWALGVLIYEMTVGFPPFYADEPIQIYEKIVSGRVRFPSKLSSDLKDLLRSLLQVDLTKRFGNLRNG<br>VGDIKNHKWFATTSWIAIYEKKVEAPFIPKYTGPGDASNFDDYEEEELRISINEKCAKEFSEF
M. mulatta	>ENSMMUP00000004604_Macaca_mulatta (b)<br>SVKEFLAKAKEDFLKKWENPTQNNAGLEDFERKKTLGTGSFGRVMLVKHKATEQYYAMKILDKQKVVKLKQI<br>EHTLNEKRILQAVNFPFLVRLEYAFKDNSNLYMVMEYVPGGEMFSHLRRIGRFSEPHARFYAAQIVLTFEYL<br>HSLDLIYRDLKPENLLIDHQGYIQVTDFGFAKRVKGRTWTLCGTPEYLAPEIILSKGYNKAVDWWALGVLIY<br>EMAAGYPPFFADQPIQIYEKIVSGKVRFPSHFSSDLKDLLRNLLQVDLTKRYGNLKNGVSDIKTHKWFATTD<br>WIAIYQRKVEAPFIPKFRGSGDTSNFDDYEEEDIRVSITEKCAKEFGEF<br><br>>ENSMMUP00000026815_Macaca_mulatta (g)<br>MGNAAAKKDTEQETVNEFLAKARGDFLYRWGNPAQNTASSDQFERLKTLGTGSYGRVMLVRHRETGNHYAMK<br>ILDKQKVVRLKQVEHTLNEKRILQAINFPFLVKLQFSFKDNSNLYLVMEYVPGGEMFSHLRRVGRFSEPQAC<br>FYAAQVVLAFQYLHSLDLIHRDLKPENLLIDQQGYLQVTDFGFAKRVKGRTWTLCGTPEYLAPEIILSKGYN<br>KAVDWWALGVLIYEMTAGFPPFYADQPIQIYEKIVSGKVRFPSQLSPDLKDLLRSLLQVDLTKRFGNLKNGV<br>GDIKNHKWFATTNWIALFEKKVEAPFIPKYTGPGDASNFDSYEEEEIRISVNEKCASKFAEF
P. hamadryas	>ENSP00000366488_Papio_hamadryas (g)<br>MGNAAAKKDTEQESVNEFLAKARGDFLYRWGNPAQNTASSDQFERLKTLGTGSYGRVMLVRHRETGNHYAMK<br>ILDKQKVVRLKQVEHTLNEKRILQAINFPFLVKLQFSFKDNSNLYLVMEYVPGGEMFSHLRRVGRFSEPQAC<br>FYAAQVVLAFQYLHSLDLIHRDLKPENLLIDQQGYLQVTDFGFAKRVKGRTWTLCGTPEYLAPEIILSKGYN<br>KAVDWWALGVLIYEMTAGFPPFYADQPIQIYEKIVSGKVRFPSQLSPDLKDLLRSLLQVDLTKRFGNLKNGV<br>GDIKNHKWFATTNWIALFEKKVEAPFIPKYTGPGDASNFDDYEEEEIRISVNDKCASKFAEF

C. jacchus	>ENSCJAP00000045124_Callithrix_jacchus (b)<br>MGNAATAKKGSEVESVKEFLAKAKEDFLKKWENPTPSNAGLEDFERKKTLGTGSFGRVMLVKHKATEQYYAM<br>KILDKQKVVKLKQIEHTLNEKRILQAVNFPFLVRLEYAFKDNSNLYMVMEYVPGGEMFSHLRRIGRFSEPHA<br>RFYAAQIVLTFEYLHSLDLIYRDLKPENLLIDHQGYIQVTDFGFAKRVKGRTWTLCGTPEYLAPEIILSKGY<br>NKAVDWWALGVLIYEMAAGYPPFFADQPIQIYEKIVSGKVRFPSHFSSDLKDLLRNLLQVDLTKRFGNLKNG<br>VSDIKTHKWFATTDWIAIYQRKVEAPFIPKFRGSGDTSNFDDYEEEDIRVSITEKCGREFDEF<br><br>>ENSCJAP00000049480_Callithrix_jacchus_truncated (g)<br>RAKEDFLYRWEHPVQNTASSDQFELLKTLGAGSFGRVVLVRHQESGGHYAMKILNKQKVVKLKQVEQVLSEK<br>RILQAINFPFLVKLQFSFRDNSNLYLVMEYVPGGDMFSRLRRVGKFREPHACFCAAQIVLAFQYLHSRDLIH<br>RDLKPENIVIDQQGYLRVTDFGFAKRVRGRTWTLCGTPEYLAPEIILNKGYSKAVDWWALGVLTYEMAMGFP<br>PFYADQAVQIYQKIVSGKVQFPSHVSSDLKDLLRNLLQVDLSKRFGNLRNGVGDIKNHKWFTTTNWIAIYEK<br>KVKAPFIPKLAGPGDASNFDNYEEEEIWISINEKCANEFSEF<br><br>>ENSCJAP00000040924_Callithrix_jacchus (rt)<br>MGNVPAKDTEQKVVKEFIAGAKEDFLRRWERPVQNTARLDQFKLLKTLGIGSFGRVVLVSHRESGSHYAMKI<br>LNKEKVVRLKQVQHVLNEKRILQAIDFPFLVKLHFSFRDNSNLYLVMEYVPGGDVFSHLQRVGRFTEPHAGF<br>YAAQIVLAFQYLHSLDLIHRDLKPENIVIDQQGYLRVTDFGFAKRVRGRTWTLCGTPEYLAPEIILNKGYSK<br>AVDWWTLGVLIYEMAVGFPPFYADQRNSVFQNILSGKVRFPSHVSSDLKNLLRNLLQVDLSKRFGNLRNGVG<br>DIKNHKWFATTNWIAIYEKQVEAPFIPKFAGPGDASNFDNYEEEEIRISINEKYANEFSQF
O. garnettii	>ENSOGAP00000010597_Otolemur_garnettii (a)<br>MGNAAAAKKGSEQESVKEFLAKAKEDFLKKWENPAQNTAHLDHFERIKTLGTGSFGRVMLVRHKETGNHYAM<br>KILDKQKVVKLKQIEHTLNEKRILQAVNFPFLVRLEFSFKDNSNLYMVMEYVPGGEMFSHLRRIGRFSEPHA<br>RFYAAQIVLTFEYLHSLDLIYRDLKPENLLIDQQGYIQVTDFGFAKRVKGRTWTLCGTPEYLAPEIILSKGY<br>NKAVDWWALGVLIYEMAAGYPPFFADQPIQIYEKIVSGKVRFPSHFSSDLKDLLRNLLQVDLTKRFGNLKNG<br>VNDIKNHKWFATTDWIAIYQRKVEAPFIPKFKGPGDTSNFDDYEEEEIRVSINEKCGKEFSEF<br><br>>ENSOGAP00000005225_Otolemur_garnettii (b)<br>MAAYRELPCNQFTGTTTALQKLEGFASRLFHRHSKGADLKTALENDSLHFSEHTAVWDRSMKEFLAKAKEDF<br>LKKWENPPPNNAGLEDFERKKTLGTGSFGRVMLVKHKATEQYYAMKILDKQKVVKLKQIEHTLNEKRILQAV<br>NFPFLVRLEYSFKDNSNLYMVMEYVPGGEMFSHLRRIGRFSEPHARFYAAQIVLTFEYLHSLDLIYRDLKPE<br>NLLIDHQGYIQVTDFGFAKRVKGRTWTLCGTPEYLAPEIILSKGYNKAVDWWALGVLIYEMAAGYPPFFADQ<br>PIQIYEKIVSGKVRFPSHFSSDLKDLLRNLLQVDLTKRFGNLKNGVSDIKTHKWFATTDWIAIYQRKVEAPF<br>IPKFRGSGDTSNFDDYEEEDIRVSITEKCAKEFGEF
T. syrichta	>ENSTSYP00000000346_Tarsius_syrichta (b)<br>MAAYRELPCNQYTGTTTALQKLEGFASRLFHRHSKGTAHDQKTALENDSLHFSEHTASWDRSMKEFLAKAKE<br>DFLKKWENPAPNNAGLEDFERKKTLGTGSFGRVMLVKHKATEQYYAMKILDKQKVVKLKQIEHTLNEKRILQ<br>AVNFPFLVRLEYSFKDNSNLYMVMEYVPGGEMFSHLRRIGRFSEPHARFYAAQIVLTFEYLHSLDLIYRDLK<br>PENLLIDHQGYIQVTDFGFAKRVKGRTWTLCGTPEYLAPEIILSKGYNKAVDWWALGVLIYEMAAGYPPFFA<br>DQPIQIYEKIVSGKVRFPSHFSSDLKDLLRNLLQVDLTKRFGNLKNGVSDIKTHKWFATTDWIAIYQRKVEA<br>PFIPKFRGSGDTSNFDDYEEEDIRVSITEKCGKEFGEF
S. scrofa	>ENSSSCP00000014641_Sus_scrofa (a)<br>MGNAAAAKKGSEQESVKEFLAKAKEDFLKKWENPAQNTAHLDQFERIKTLGTGSFGRVMLVKHKETGNHFAM<br>KILDKQKVVKLKQIEHTLNEKRILQAVNFPFLVKLEFSFKDNSNLYMVMEYVPGGEMFSHLRRIGRFSEPHA<br>RFYAAQIVLTFEYLHSLDLIYRDLKPENLLIDQQGYIQVTDFGFAKRVKGRTWTLCGTPEYLAPEIILSKGY<br>NKAVDWWALGVLIYEMAAGYPPFFADQPIQIYEKIVSGKVRFPSHFSSDLKDLLRNLLQVDLTKRFGNLKNG<br>VNDIKNHKWFATTDWIAIYQRKVEAPFIPKFKGPGDTSNFDDYEEEEIRVSINEKCGKEFSEF
D. ordii	>ENSDORP00000014055_Dipodomys_ordii (a)<br>MGNAAAAKKGSEQESVKEFLAKAKEDFLKKWENPAQNTAHLDQFDRIKTLGTGSFGRVMLVKHKETGNHYAM<br>KILDKQKVVKLKQIEHTLNEKRILQAVNFPFLVKLEFSFKDNTNLYMVMEYVPGGEMFSHLRRIGRFSEPHA<br>RFYAAQIVLTFEYLHSLDLIYRDLKPENLLIDQQGYIQVTDFGFAKRVKGRTWTLCGTPEYLAPEIILSKGY<br>NKAVDWWALGVLIYEMAAGYPPFFADQPIQIYEKIVSGKVRFPSHFSSDLKDLLRNLLQVDLTKRFGNLKNG<br>VNDIKNHKWFATTDWIAIYQRKVEAPFIPKFKGAGDTSNFDDYEEEEIRISINEKCGKEFSEF
C. porcellus	>ENSCPOP00000016885_Cavia_porcellus (a)<br>SVKEFLAKAKEDFLKKWESPAQNTASLDQFDRIKTLGTGSFGRVMLVKHKETGNHYAMKILDKQKVVKLKQV<br>EHTLNEKRILQAVNFPFLVRLEYSFKDNSNLYMVMEYVPGGEMFSHLRRIGRFSEPHARFYAAQIVLTFEYL<br>HSLDLIYRDLKPENLLIDHQGYIQVTDFGFAKRVKGRTWTLCGTPEYLAPEIILSKGYNKAVDWWALGVLIY<br>EMAAGYPPFFADQPIQIYEKIVSGKVRFPSHFSSDLKDLLRNLLQVDLTKRFGNLKNGVNDIKNHKWFATTD<br>WIAIYQRKVEAPFIPKFKGAGDTSNFDDYEEEEIRVSINEKCGKEFSEF<br><br>>ENSCPOP00000008705_Cavia_porcellus (b)<br>LRNSYMTKPFSYMFSVKEFLAKAKEDFLKKWENPPPNNAGLEDFERKKTLGTGSFGRVMLVKHKATEQYYAM<br>KILDKQKVVKLKQIEHTLNEKRILQAVNFPFLVRLEYSFKDNSNLYMVMEYVPGGEMFSHLRRIGRFSEPHA<br>RFYAAQIVLTFEYLHSLDLIYRDLKPENLLIDHQGYIQVTDFGFAKRVKGRTWTLCGTPEYLAPEIILSKGY<br>NKAVDWWALGVLIYEMAAGYPPFFADQPIQIYEKIVSGKVRFPSHFSSDLKDLLRNLLQVDLTKRFGNLKNG<br>VSDIKTHKWFATTDWIAIYQRKVEAPFIPKFRGSGDTSNFDDYEEEDIRVSITEKCSKEFSDF
O. cuniculus	>ENSOCUP00000003134_Oryctolagus_cuniculus (b)<br>MGNAATAKKGSEVESVKEFLAKAKEDFLKKWENPPPNNAGLEDFERKKTLGTGSFGRVMLVKHKATEQYYAM<br>KILDKQKVVKLKQIEHTLNEKRILQAVNFPFLVRLEYSFKDNSNLYMVMEYVPGGEMFSHLRRIGRFSEPHA<br>RFYAAQIVLTFEYLHSLDLIYRDLKPENLLIDHQGYIQVTDFGFAKRVKGRTWTLCGTPEYLAPEIILSKGY<br>NKAVDWWALGVLIYEMAAGYPPFFADQPIQIYEKIVSGKVRFPSHFSSDLKDLLRNLLQVDLTKRFGNLKNG<br>VSDIKTHKWFATTDWIAIYQRKVEAPFIPKFRGSGDTSNFDDYEEEDIRVSITEKCAKEFCEF

E. caballus	>ENSECAP00000020287_Equus_caballus (b)<br>MSARKSSEASACSSSEVSVKEFLAKAKEDFLKKWENPAQNNAGLEDFERKKTLGTGSFGRVMLVKHKATEQY<br>YAMKILDKQKVVKLKQIEHTLNEKRILQAVNFPFLVRLEYSFKDNSNLYMVMEYVPGGEMFSHLRRIGRFSE<br>PHARFYAAQIVLTFEYLHSLDLIYRDLKPENLLIDHQGYIQVTDFGFAKRVKGRTWTLCGTPEYLAPEIILS<br>KGYNKAVDWWALGVLIYEMAAGYPPFFADQPIQIYEKIVSGKVRFPSHFSSDLKDLLRNLLQVDLTKRFGNL<br>KNGVSDIKTHKWFATTDWIAIYQRKVEAPFIPKFRGSGDTSNFDDYEEEDIRVSITEKCAKEFCEF
P. vampyrus	>ENSPVAP00000016560_Pteropus_vampyrus (a)<br>MGNAAAAKKGSEQESVKEFLAKAKEDFLKKWENPAQNTAHLDQFERIKTLGTGSFGRVMLVKHKETGSHFAM<br>KILDKQKVVKLKQIEHTLNEKRILQAVNFPFLVRLEFSFKDNSNLYMIMEYVPGGEMFSHLRRIGRFSEPHA<br>RFYAAQIVLTFEYLHSLDLIYRDLKPENLLIDQQGYIQVTDFGFAKRVKGRTWTLCGTPEYLAPEIILSKGY<br>NKAVDWWALGVLIYEMAAGYPPFFADQPIQIYEKIVSGKVRFPSHFSSDLKDLLRNLLQVDLTKRFGNLKNG<br>VNDIKNHKWFATTDWIAIYQRKVEAPFIPKFKGPGDTSNFDDYEEEEIRVSINEKCGKEFSEF
T. truncatus	>ENSTTRP00000013926_Tursiops_truncatus (a)<br>MGNAAAPKKGSEQESVKEFLAKAKEDFLKKWENPAQNTAHLDQFERIKTLGTGSFGRVMLVKHKETGNHFAM<br>KILDKQKVVKLKQIEHTLNEKRILQAVNFPFLVKLEFSFKDNSNLYMIMEYVPGGEMFSHLRRIGRFSEPHA<br>RFYAAQIVLTFEYLHSLDLIYRDLKPENLLIDQQGYIQVTDFGFAKRVKGRTWTLCGTPEYLAPEIILSKGY<br>NKAVDWWALGVLIYEMAAGYPPFFADQPIKIYDKIVSGKVRFPSHFSSDLKDLLRNLLQVDLTKRFGNLKNG<br>VNDIKNHKWFATTDWIAIYQRKVEAPFIPKFKGPGDTSNFDDYEEEEIRVSISEKCGKEFSEF<br><br>>ENSTTRP00000010712_Tursiops_truncatus (b)<br>MAAYRELPCNQYTGTTALQKLEGFASRLFHRHSKGTAHDQKTTLENDSLHFSEHTALWDRSMKEFLAKAKED<br>FLKKWENPAPNNAGLEDFERKKTLGTGSFGRVMLVKHKATEQYYAMKILDKQKVVKLKQIEHTLNEKRILQA<br>VNFPFLVRLEYSFKDNSNLYMVMEYVPGGEMFSHLRRIGRFSEPHARFYAAQIVLTFEYLHSLDLIYRDLKP<br>ENLLIDHQGYIQVTDFGFAKRVKGRTWTLCGTPEYLAPEIILSKGYNKAVDWWALGVLIYEMAAGYPPFFAD<br>QPIQIYEKIVSGKVRFPSHFSSDLKDLLRNLLQVDLTKRFGNLKNGVSDIKTHKWFATTDWIAIYQRKVEAP<br>FIPKFRGSGDTSNFDDYEEEDIRVSITEKCGKEFCEF
M. eugenii	>ENSMEUP00000001850_Macropus_eugenii (a)<br>KEFLAKAKEEFLKKWESPPQSTVQLDQFDRIKTLGTGSFGRVILVKHRETGNYFAMKVLDKQKVVKLKQIEH<br>TMNEKRILQAINFPFLVRLEYSFKDNTNLYMVMEYVPGGEMFSHLRRIGRFSEPHARFYAAQIVLTFEYLHS<br>LDLIYRDLKPENLLIDQQGYIQVTDFGFAKRVKGRTWTLCGTPEYLAPEIILSKGYNKAVDWWALGVLIYEM<br>AAGYPPFFADQPIQIYEKIVSGKVRFPSHFSSDLKDLLRNLLQVDLTKRYGNLKNGVNDIKNHKWFATTDWI<br>AIYQRK<br><br>>ENSMEUP00000011811_Macropus_eugenii (rt)<br>MGNAATAKKGSELESVKEFLAKAKEEFLKKWEAPAQNTAQLEHFDRIKTLGTGSFGRVMLVKHKETGNHFAM<br>KILDKQKVVKLKQIEHTLNEKRILQAVNFPFLVRLEYSFKDNTNLYMVMEYVLGGEMFSHLRRIGRFSEPHA<br>RFYAAQIVLTFEYLHSLDLIYRDLKPENLLIDQHGYIQVTDFGFAKRVKGRTWTLCGTPEYLAPEIILSKGY<br>NKAVDWWALGVLIYEM
M. domestica	>ENSMODP00000015141_Monodelphis_domestica (a)<br>SVKEFLAKAKEEFLKKWENPSQPSVIQLDLYERLKTLGTGSFGRVVLVKSKETGNFFAMKILDKQKVVKLKQ<br>IEHTMNEKRILQSISFPFLVKLECAFKDNSNLYMVMEYVAGGEMFSHLRRIGRFSEPHARFYAAQIVLTFEY<br>LHSLDLIYRDLKPENLLIDQQGYIQVTDFGFAKRVKGRTWTLCGTPEYLAPEIILSKGYNKAVDWWALGVLI<br>YEMAAGYPPFFADQPIQIYEKIVSGKVRFPSHFSSDLKDLLRNLLQVDLTKRYGNLKNGVNDIKNHKWFATT<br>DWIAIYQRKVEAPFIPKCKGPGDTSNFDDYEEEELRISISEKCPKEFAEF
A. carolinensis	>ENSACAP00000005713_Anolis_carolinensis (b)<br>MAAQKELQGNPSTGTANALQRLEGFANRLFHRHSKGSSSELKAAQENESPHFSELAALWERSMKEFLAKAKE<br>DFLRKWESPPQNTAGLEDFERLKTLGTGSFGRVMLVKHKATEQYYAMKILDKQKVVKLKQIEHTLNEKRILQ<br>AVNFPFLVRLEYSFKDNSNLYMVMEYVPGGEMFSHLRRIGRFSEPHARFYAAQIVLTFEYLHSLDLIYRDLK<br>PENLLIDQQGYIQVTDFGFAKRVKGRTWTLCGTPEYLAPEIILSKGYNKAVDWWALGVLIYEMAAGYPPFFA<br>DQPIQIYEKIVSGKVRFPSHFSSDLKDLLRNLLQVDLTKRYGNLKNGVNDIKNHKWFATTDWIAIYQRKVEA<br>PFIPKCRGPGDTSNFDDYEEEDIRVSLTEKCAKEFADF

T. rubripes	>ENSTRUP00000013710_Takifugu_rubripes (a)<br>MGNAPTARKGSEMESVKEFLAKAKEDFLKKWENPAQNTACLEQFERLKTLGTGSFGRVMLVKHRETGQHYAM<br>KILNKQKVVKLKQIEHTLNEKRILQAVSFPFLVRLEYSFKDNTNLYMVMEYVPGGEMFSHLRRIGRFSEPHA<br>RFYAAQIVLTFEYLHALDLIYRDLKPENLLIDQQGYIQVTDFGFAKRVKGRTWTLCGTPEYLAPEIILSKGY<br>NKAVDWWALGVLVYEMAAGYPPFFADQPIQIYEKIVSGKVRFPSHFSSDLKDLLRNLLQVDLTKRYGNLKNG<br>VNDIKGHKWFATTDWIAIYQKKVEAPFIPKFKGPGDTSNFDDYEEEEIRVSFTEKCAKEFAEF<br><br>>ENSTRUP00000032221_Takifugu_rubripes (a)<br>MGNTPTTKKGNEMESVKEFLAKAKEDFLKKWENPAQQTAALDHFERLKTLGTGSFGRVMLVKHKESGQHFAM<br>KILDKQKVVKLKQIEHTLNEKRILQAVNFPFLVCLEHSFKDNSNLYMVMEYVPGGEMFSHLRRIGRFSEPHA<br>RFYAAQIVLTFEYLHSLDLIYRDLKPENLLIDQQGYIQVTDFGFAKRVKGRTWTLCGTPEYLAPEIILSKGY<br>NKAVDWWALGVLIYEMAAGYPPFFADQPIQIYEKIVSGKVRFPSHFSSDLKDLLRNLLQVDLTKRFGNLRNG<br>VNDIKGHKWFATTDWIAIYQRKVEAPFIPKCKGPGDTSNFDDYEEEEIRVSITEKCAKEFAEF<br><br>>ENSTRUP00000015108_Takifugu_rubripes (b)<br>MGNAATAKKGNEQESVKEFLAKAKEDFLRKWECPQQCTTGLDDFDRFKTLGTGSFGRVMLVKHKETNQFYAM<br>KILDKQKVVKLKQIEHTLNEKRILQAVSFPFLVRLVYSFKDNSNLYMVMEYVPGGEMFSHLRRIGRFSEHHA<br>RFYAAQIILTFEYLHSLDLIYRDLKPENLLIDQQGYIQVTDFGFAKRVKGRTWTLCGTPEYLAPEIILSKGY<br>NKAVDWWALGVLIYEMAAGYPPFFADQPIQIYEKIVSGKVRFPSHFSSDLKDLLRNLLQVDLTKRFGNLKNG<br>VNDIKNHKWFSTTDWIAIYEKKVEAPFLPKCRGPGDTSNFDDYEEEDVHVSQTEKCAKEFADF<br><br>>ENSTRUP00000026750_Takifugu_rubripes (b)<br>MGNAATAKKGNELESVKEFLAKAKEDFLRKWECPPQSTTCLDDFDRLKTLGTGSFGRVMLVKHKGTEQYFAM<br>KILDKQKVVKLKQIEHTLNEKRILQAVSFPFLVRLEYAFKDNSNLYMVMEYVPGGEMFSHLRRIGRFSEPHA<br>RFYAAQIVLTFEYLHSLDLIYRDLKPENLLIDHHGYIQVTDFGFAKRVKGRTWTLCGTPEYLAPEIILSKGY<br>NKAVDWWALGVLIYEMAAGYPPFFADQPIQIYEKIVSGKVRFPSHFSSDLKDLLRNLLQVDLTKRYGNLKNG<br>VNDIKGHKWFATTDWIAIYERKVEAPFIPKCRGPGDTSNFDDYEEEEIRVSLTEKCAKEFAEF
O. latipes	>ENSORLP00000008832_Oryzias_latipes (a)<br>AAIFTHNMFLFAVKEFLAKAKEDFLKKWENPAQQTAALDHFERLKTLGTGSFGRVMLVKHKETGQHFAMKIL<br>DKQKVVKLKQIEHTLNEKRILQAVSFPFLVQLEHSFKDTSNLYMVMEYVPGGEMFSHLRRIGRFSEPHARFY<br>AAQIVLTFEYLHSLDLIYRDLKPENLLIDQQGYIQVTDFGFAKRVKGRTWTLCGTPEYLAPEIILSKGYNKA<br>VDWWALGVLIYEMAAGYPPFFADQPIQIYEKIVSGKVRFPSHFSSDLKDLLRNLLQVDLTKRFGNLRNGVND<br>IKGHKWFATTDWIAIYQRKVEAPFIPKCKGPGDTSNFDDYEEEEIRVSFSEKCAKEFAEF<br><br>>ENSORLP00000018527_Oryzias_latipes_truncated (a)<br>VKEFLAKAKEDFLKKWENPSQNTANLEQFERLKTLGTGSFGRVMLVKHRETGQHYAMKILNKQKVVKLKQIE<br>HTLNEKRILQAVSFPFLVRLEFSFKDNTNLYMVMEYVPGGEMFSHLRKIGRFSEPHARFYATQIVLTFEYLH<br>ALDLIYRDLKPENLLIDQQGYIQVTDFGFAKRVKGRTWTLCGTPEYLAPEIILSKGYNKAVDWWALGVLVYE<br>MAAGYPPFFADQPIQIYEKIVSGKVRFPSHFSSDLKDLLRNLLQVDLTKRYGNLKNGVNDIKGHKWFAATDW<br>IAIFQKKVEAPFVPKFKGPGDTSNFDDYEEEEIRVSFSEKCAKEFAEF<br><br>>ENSORLP00000007943_Oryzias_latipes (b)<br>MGNTATAKKGNEQESVKEFLAKAKEDFLRKWECPPQCTTSLDDFERFKTLGTGSFGRVMLVKHKATNQFYAM<br>KILDKQKVVKLKQIEHTLNEKRILQAVSFPFLVRLVYAFKDNSNLYMVMEYVPGGEMFSHLRRTGRFSEHHA<br>RFYAAQIVLTFEYLHSLDLIYRDLKPENLLIDQHGYIQVTDFGFAKKVKGRTWTLCGTPEYLAPEIILSKGY<br>NKAVDWWALGVLIYEMAAGYPPFFADQPIQIYEKIVSGKVRFPSHFSSDLKDLLRNLLQVDLTKRFGNLKNG<br>VNDIKNHKWFSTTDWIAIYERKIEAPFLPKCRGPGDTSNFDDYEEEDIHVSQTEKCGKEFADF<br><br>>ENSORLP00000021647_Oryzias_latipes (b)<br>MGNAATAKKGNELESVKEFLAKAKEDFLRKWECPPQSTTCLEDFDRIKTLGTGSFGRVMLVKHKSSEQYFAM<br>KILDKQKVVKLKQIEHTLNEKRILQAVSFPFLVRLEYAFKDNSNLYMVMEYVPGGEMFSHLRRIGRFSEPHA<br>RFYAAQIVLTFEYLHSLDLIYRDLKPENLLIDHHGYIQVTDFGFAKRVKGRTWTLCGTPEYLAPEIILSKGY<br>NKAVDWWALGVLIYEMAAGYPPFFADQPIQIYEKIVSGKVRFPSHFSSDLKDLLRNLLQVDLTKRYGNLKNG<br>VNDIKGHKWFATTDWIAIYERKVEAPFIPKCRGPGDTSNFDDYEEEEIRVSLTEKCAKEFAEF
G. aculeatus	>ENSGACP00000013822_Gasterosteus_aculeatus (a)<br>MGNTPTAKKGNEMESVKEFLAKAKEDFLKKWENPAQQTAALDHFERLKTLGTGSFGRVMLVKHKESGQHFAM<br>KILDKQKVVKLKQIEHTLNEKRILQAVSFPFLVRLEHSFKDNSNLYMIMEYVPGGEMFSHLRRIGRFSEPHA<br>RFYAAQIVLTFEYLHSLDLIYRDLKPENLLIDQQGYIQVTDFGFAKRVKGRTWTLCGTPEYLAPEIILSKGY<br>NKAVDWWALGVLIYEMAAGYPPFFADQPIQIYEKIVSGKVRFPSHFSSDLKDLLRNLLQVDLTKRFGNLRNG<br>VNDIKGHKWFATTDWIAIYQRKVEAPFIPKCKGPGDTSNFDDYEEEEIRVSFTEKCAKEFAEF<br><br>>ENSGACP00000024909_Gasterosteus_aculeatus_fixedExon5 (a)<br>MGNAPTARKGSEMESVKEFLAKAKEDFLKKWESPAQNTAGLEQFERLKTLGTGSFGRVMLVKHRETGQHYAM<br>KILNKQKVVKLKQIEHTLNEKRILQAVSFPFLVRLEYSFKDNTNLYMVMEYVPGGEMFSHLRRIGRFSEPHA<br>RFYAAQIVLTFEYLHALDLIYRDLKPENLLIDQQGYIQVTDFGFAKRVKGRTWTLCGTPEYLAPEIILSKGY<br>NKAVDWWALGVLVYEMAAGYPPFFADQPIQIYEKIVSGKVRFPSHFSSDLKDLLRNLLQVDLTKRFGNLKNG<br>VNDIKGHKWFATTDWIAIYQKKVEAPFVPKFKGPGDTSNFDDYEEEEIRVSFNEKCGKEFAEF<br><br>>ENSGACP00000023579_Gasterosteus_aculeatus (b)<br>MGNAATAKKGNELESVKEFLAKAKEDFLRKWECPPQSATCLDDFDRLKTLGTGFLWRVMLVKHKATEQHFAM<br>KILDKQKVVKLKQIEHTLNEKRILQAVSFPFLVKLEYAFKDTSNLYMVMEYVPGGEMFSHLRRIGRFSEPHA<br>RFYAAQIVLTFEYLHSLDLIYRDLKPENLLIDHHGYIQVTDFGFAKRVKGRTWTLCGTPEYLAPEIILSKGY<br>NKAVDWWALGVLIYEMAAGYPPFFADQPIQIYEKIVSGKVRFPSHFSSDLKDLLRNLLQVDLTKRYGNLKNG<br>VNDIKGHKWFATTDWIAIYERKVEAPFIPKCRGPGDTSNFDDYEEEEIRVSVTEKCGKEFAEF<br><br>>ENSGACP00000012693_Gasterosteus_aculeatus (b)<br>MGNAATAKKGNEQESVKEFLAKAKEDFLRKWECPPQSTTGLDDFDRFKTLGTGSFGRVMLVKHKESNQFYAM<br>KILDKQKVVKLKQIEHTLNEKRILQAVSFPFLVRLEYSFKDNSNLYMVMEYVPGGEMFSHLRRIGRFSEHHA<br>RFYAAQIVLTFEYLHSLDLIYRDLKPENLLIDQHGYIQVTDFGFAKRVKGRTWTLCGTPEYLAPEIILSKGY<br>NKAVDWWALGVLIYEMAAGYPPFFADQPIQIYEKIVSGKVRFPSHFSSDLKDLLRNLLQVDLTKRFGNLKNG<br>VNDIKNHKWFSQTDWIAIYERKVEAPFLPKCRGPGDTSNFDDYEEEDIRVSQTEKCAKEFAEF

T. nigroviridis	>ENSTNIP00000013350_Tetraodon_nigroviridis (a)<br>MGNTPTTKKGNEMESVKEFLAKAKEDFLKKWENPAQQTAALDHFDRLKTLGTGSFGRVMLVKHKETGQHFAM<br>KILDKQKVVKLKQIEHTLNEKRILQAVNFPFLVCLEHSFKDNSNLYMVMEYVPGGEMFSHLRRIGRFSEPHA<br>RFYAAQIVLTFEYLHSLDLIYRDLKPENLLIDQQGYIQVTDFGFAKRVKGRTWTLCGTPEYLAPEIILSKGY<br>NKAVDWWALGVLIYEMAAGYPPFFADQPIQIYEKIVSGKVRFPSHFSSDLKDLLRNLLQVDLTKRFGNLRNG<br>VNDIKGHKWFATTDWIAIYQRKVEAPFIPKCKGPGDTSNFDDYDEEEIRVSFTEKCAKEFAEF<br><br>>ENSTNIP00000015136_Tetraodon_nigroviridis (b)<br>VSPVKEFLAKAKEDFLRKWECPQRCTTGLDDFDRFKTLGTGSFGRVMLVKHKETNQFYAMKILDKQKVVKLK<br>QIEHTLNEKRILQAVSFPFLVRLVYAFKDNSNLYMVMEYVPGGEMFSHLRRIGRFSEQHARFYAAQIILTFE<br>YLHSLDLIYRDLKPENLLIDQQGYIQVTDFGFAKRVKGRTWTLCGTPEYLAPEIILSKGYNKAVDWWALGVL<br>IYEMAAGYPPFFADQPIQIYEKIVSGKVRFPSHFSSDLKDLLRNLLQVDLTKRFGNLKNGVNDIKNHKWFST<br>TDWIAIYEKKVEAPFLPKCRGPGDTSNFDDYEEEDVHISQTEKCAKEFDDF<br><br>>ENSTNIP00000011358_Tetraodon_nigroviridis (b)<br>MGNAATAKKGNELESVKEFLAKAKEDFLRKWECPPQSTTCLDDFDRLKTLGTGSFGRVMLVKHKGTEQYFAM<br>KILDKQKVVKLKQIEHTLNEKRILQAVSFPFLVKLEYAFKDNSNLYMVMEYVPGGEMFSHLRRIGRFSEPHA<br>RFYAAQIVLTFEYLHSLDLIYRDLKPENLLIDHHGYIQVTDFGFAKRVKGRTWTLCGTPEYLAPEIILSKGY<br>NKAVDWWALGVLIYEMAAGYPPFFADQPIQIYEKIVSGKVRFPSHFSSDLKDLLRNLLQVDLTKRYGNLKNG<br>VNDIKGHKWFATTDWIAIYERKVEAPFIPKCRGPGDTSNFDDYEEEEIRVSLTEKCAKEFAEF
C. intestinalis	>ENSCINP00000013406_Ciona_intestinalis<br>MGNTPAKKGDQAESVKAFLAEAKEEFLKKWENPIQNNSKLDDFERLKTLGTGSFGRVILVKRKENSNYYAMK<br>ILDKQKVVKLKQVEHTLNEKKILQAINFPFLVKMDFSFKDNSNLYMVLEYVIGGEMFSHLRRIGRFSESHSR<br>FYAAQIVLGFEYLHYLDIIYRDLKPENLLIDQQGYIQVTDFGFAKRVKGRTWTLCGTPEYLAPEIILSKGYN<br>KAVDWWALGVLIYEMAAGYPPFFADQPIQIYEKIVSGKVRFPSHFTSELKDLLRNLLQVDLTKRFGNLKNGV<br>SDIKGHRWFQPIDWIAVYQRKLDAPFIPKFKHPGSTENFDDYEEEQLRVHSTEKCVKEFADF
C. savignyi	>Homolog1_Ciona_savignyi<br>MGNTPAKKGDQAESVKAFLAEAKEEFLKKWENPIQNNSKLDDFERLKTLGTGSFGRVILVKRKENTSFYAMK<br>ILDKQKVVKLKQVEHTLNEKKILQAINFPFLVKMDFSFKDNSNLYMVLEYVIGGEMFSHLRRIGRFSESHSR<br>FYAAQIVLGFEYLHYLDIIYRDLKPENLLIDQQGYIQVTDFGFAKRVKGRTWTLCGTPEYLAPEIILSKGYN<br>KAVDWWALGVLIYEMAAGYPPFFADQPIQIYEKIVSGKVRFPSHFTSELKDLLRNLLQVDLTKRFGNLKNGV<br>ADIKGHRWFQPIDWIAVYQKKLEAPFMPKFKHPGSTENFDDYEEEQLRIHSTEKCVKEFADF
P. marinus	>Homolog1_Petromyzon_marinus<br>KEFLAKAKEDFMRKWENPQQNTSCLEDFERIKTLGTGSFGRVMLVKHKSSDQFFAMKILDKQKVVKLKQVEH<br>TLNEKRILQAISFPFLVRLEYSFKDNSNLYMVLEYVPGGEMFSHLRRIGRFSEPHSRFYAAQIVLAFEYLHS<br>LDLIYRDLKPENLLIDQQGYIQVTDFGFAKRVKGRTWTLCGTPEYLAPEIILSKGYNKAVDWWALGVLIYEM<br>AAGYPPFFADQPIQIYEKIVSGKVRFPSHFSSDLKDLLRNLLQVDLTKRFGNLKNGVNDIKNHKWFSTTDWI<br>AIYQRKVEAPFIPKCKGPGDASNFDDYEEEEIRISSTEKCAKEFADF<br><br>>Homolog2_Petromyzon_marinus<br>KEFLAKAKEDFLKKWENPQQNTSCLDDFERMKTLGTGSFGRVMLVKHKATDRYFAMKILDKQKIMRLKQVEH<br>TLNEKRILQAISFPFLVSLEYSYKDNSNLYMVLEYVPGGEMFSHLRRIGRFSEPHSRFYAAQIVLAFEYLHS<br>LDLIYRDLKPENLLIDQQGYIQXXXXXXXXXXXXXXXXXXXXXXXXXXXXXXXXGYNKAVDWWALGVLVYEM<br>AAGYPPFFADQPIQIYEKIVSGK
S. acanthias	>Homolog1_Squalus_acanthias<br>MGNAATAKKGNEIESVKEFLAKAKEDFLRKWESPPQNTAGLDDFERQKTLGTGSFGRVMLVKHKGTEQYYAM<br>KILDKQKVVKLKQIEHTLNEKRILQAVNFPFLVKLEYSFRDNSNLYM<br><br>>Homolog2_Squalus_acanthias<br>KSHRKKNPTDLCFKIMNQCSEVTCQQAADHKGSKMSSALQKLENLAIRLFNRSTRGSPQDFETGSESDIRPE<br>SEYCLAWNLHMKDFLAKAKEEFLEKWESPTQNTASLDDFERTKTLGTGSFGRVMLVKHKITKEHYAMKILDK<br>QKVVKLKQIEHTLNEKKILQAVRFPFLVRLVFTFKDNTNLYMVMEYIQGGEMFSHLRRSGRFSEPHARFYAA<br>QIVLAFEYLHSLDLVYRDLKP
L. erinacea	>Homolog1_Leucoraja_erinacea<br>MKLWRLTSREWNKDSNSSSPSIVYKDHHPKNQEIETVKDYLEKAREEFLQKWESPSQNTASLDDFDRTKTLG<br>TGSFGRVMLVKHKATKEHYAMKILDKQKVVKLKQIEHTLNEKRILQAVRFPFLVRLVFSFKDNTNLYMVMEY<br>IQGGEMFSHLRRSGRFSEPHSRFYAAQIVLAFEYLHSLDLLYRDLKPENILN<br><br>>Homolog2_Leucoraja_erinacea<br>MKLWRLTSREWNKDSNSSSPSIVYKDHHPKNQEIETVKDYLEKAREEFLQKWESPSQNTASLDDFDRTKTLG<br>TGSFGRVMLVKHRATKEHYAMKILDKQNVVQLKQIEHTLNEKRILQAVRFPFLVRLVFSFQDNTNLYMAME
