# Supplementary material for: Evolutionary Paths of the cAMP-Dependent Protein Kinase (PKA) Catalytic Subunits
Source: PLoS One. 2013 Apr 12;8(4):e60935. doi: 10.1371/journal.pone.0060935 (PMC3625193; doi:10.1371/journal.pone.0060935)
Supplement: Materials and Methods S1 — Details on sequence data collection. (PDF) [file pone.0060935.s003.pdf]

MATERIALS AND METHODS S1

Sequence data collection. Database resources provided by UniProt (http://www.uniprot.org), the NCBI (http://www.ncbi.nlm.nih.gov), and the Ensembl project (http://www.ensembl.org) were searched for homologs of the catalytic subunits, Cα and Cβ of human PKA. The major fraction of extracted data, in particular data from Ensembl, is due to automatically predicted and annotated protein coding genes based on genomic data and a limited amount of available mRNA sequences. The underlying genomic data is of variable coverage and quality, especially for organisms for which the sequencing projects are at an early stage. Consequently, many predicted protein coding genes have missing or wrongly predicted exons, erroneous intron/exon boundaries, or predicted splice variants that are without experimental support. To complicate matters further, mammalian Cα, and in particularly Cβ, displays a large number of splice variants. It was therefore necessary to clean up the sequence data manually in order to provide reliable sequence data for further analysis. Details about the extraction and manipulation of the sequence data is given below. All sequences not readily available from sequence databases are given in Supplementary Table S1. Only a single splice variant is shown for each gene. When sufficient data is available, the displayed splice variant corresponds to human Cα1 or Cβ1. When applicable, sequences have been denoted (a), (b), (g), and (rt) for Cα, Cβ, Cγ, or other retroposon, respectively.

- Sequences for human Cα1, Cβ1, and Cγ, all with 351 residues (unprocessed, including N-terminal methionine), were obtained from UniProt with accession numbers P17612 (a), P22694 (b), and P22612 (g), respectively.
- Homologs from chimpanzee (*Pan troglodytes*) were obtained from Ensembl (Assembly: CHIMP2.1, Mar 2006. Genebuild Ensembl: Jul 2008) with identifiers ENSPTRP00000018062 (a), ENSPTRP00000042330 (b), and ENSPTRP00000035898 (g).
- Homologs from mouse (*Mus musculus*) were obtained from UniProt with accession numbers P05132 (a) and P68181 (b).
- Homologs from rat (*Rattus norvegicus*) were obtained from UniProt with accession numbers A1L1M0 (a) and P68182 (b).
- Homologs from bovine (*Bos taurus*) were obtained from UniProt with accession numbers P00517 (a) and P05131 (b).
- Homologs from pig (*Sus scrofa*) were obtained from Ensembl (Assembly: Sscrofa9, Apr 2009. Genebuild Ensembl: Sep 2009) and UniProt with accession numbers ENSSSCP00000014641 (a) and P05383 (b), respectively.
- Homologs from dog (*Canis familiaris*) were obtained from the NCBI with accession numbers NP_001003032 (a) and XP_867543 (b).
- Homologs from Sumatran orangutan (*Pongo pygmaeus abelii*) were obtained from Ensembl (Assembly: PPYG2, Sep 2007. Genebuild Ensembl: Mar 2008) with identifiers ENSPPYP00000001399 (b) and ENSPPYP00000021585 (g).
- A homolog from the gorilla (*Gorilla gorilla*) was obtained from Ensembl (Assembly: gorGor3, Dec 2009. Genebuild Ensembl: Mar 2010) as the translation of ENSGGOT00000006754 (g).
- Homologs from macaque (*Macaca mulatta*) from Ensembl (Assembly: MMUL 1.0, Feb 2006, Genebuild: Ensembl, Aug 2006) have identifiers ENSMMUP00000004604 (b) and ENSMMUP00000026815 (g).
- A homolog from Hamadryas baboon (*Papio hamadryas*) was obtained from the Ensembl pre-build site (Assembly: Pham, Nov 2008) with identifier ENSP00000366488 (g).
- Homologs from marmoset (*Callithrix jacchus*) were obtained from Ensembl (Assembly: C_jacchus3.2.1, Jan 2010, Genebuild: Ensembl, May 2010) with identifiers ENSCJAP00000045124 (b) and ENSCJAP00000040924 (rt). The two predicted 5' exons of putative transcript ENSCJAT00000003302 were removed from ENSCJAP00000049480 (g).
- Homologs from greater galago (*Otolemur garnettii*) from Ensembl (Assembly: OtoGar3, Mar 2011, Genebuild Ensembl, Dec 2011) have identifiers ENSOGAP00000010597 (a) and ENSOGAP00000005225 (b).
- A homolog from the tarsier (*Tarsius syrichta*) was obtained from Ensembl (Assembly: tarSyr1, Jul 2008. Genebuild Ensembl: Feb 2009) with identifier ENSTSYP00000000346 (b).
- Homologs from Guinea Pig (*Cavia porcellus*) were obtained from Ensembl (Assembly: cavPor3, Mar 2008. Genebuild: Ensembl, Sep 2008) with identifiers ENSCPOP00000016885 (a) and ENSCPOP00000008705 (b).
- Homologs from horse (*Equus caballus*) were obtained from the NCBI and Ensembl (Assembly: Equ Cab 2, Sep 2007. Genebuild: Ensembl, Mar 2008) with identifiers XP_001914812 (a) and ENSECAP00000020287 (b), respectively.
- Homologs from Chinese hamster (*Cricetulus griseus*) were obtained from UniProt with accession numbers P25321 (a) and P68180 (b).
- A homolog from megabat (*Pteropus vampyrus*) was obtained from Ensembl (Assembly: pteVam1, Jul 2008. Genebuild Ensembl: Feb 2009) with identifier ENSPVAP00000016560 (a).

- A homolog from sheep (*Ovis aries*) was obtained from UniProt with accession number Q9MZD9 (a).
- Homologs from rabbit (*Oryctolagus cuniculus*) were obtained from UniProt and Ensembl (Assembly: oryCun2, Nov 2009. Genebuild Ensembl: Mar 2010) with identifiers Q95J97 (a) and ENSOCUP00000003134 (b), respectively.
- A homolog from kangaroo rat (*Dipodomys ordii*) was obtained from Ensembl (Assembly: dipOrd1, Jul 2008. Genebuild Ensembl: Feb 2009) with identifier ENSDORP00000014055 (a).
- Homologs from bottlenose dolphin (*Tursiops truncatus*) from Ensembl (Assembly: turTru1, Jul 2008. Genebuild: Ensembl, Feb 2009) have identifiers ENSTTRP00000013926 (a) and ENSTTRP00000010712 (b).
- Homologs from wallaby (*Macropus eugenii*) were obtained from Ensembl (Assembly: Meug_1.0, Dec 2008. Genebuild Ensembl: Jun 2009) with identifiers ENSMEUP00000001850 (a) and ENSMEUP00000011811 (rt).
- Homologs from opossum (*Monodelphis domestica*) were obtained from Ensembl (Genebuild Ensembl: Apr 2007) and the NCBI (Assembly: monDom5, Oct 2006) with identifiers ENSMODP00000015141 (a), XP_001362513 (b), and XP_001381342 (rt). The 187 N-terminal residues of computationally predicted XP_001381342 were removed.
- Homologs from zebra finch (*Taeniopygia guttata*) and chicken (*Gallus gallus*) were obtained from the NCBI with accession numbers XP_002187574 (b) and XP_422379 (b), respectively.
- A homolog from the green anole lizard (*Anolis carolinensis*) was obtained from Ensembl (Assembly: AnoCar1.0, Feb 2007. Genebuild Ensembl: Feb 2009) with identifier ENSACAP00000005713 (b).
- Homologs from the frog *Xenopus tropicalis* were obtained from the NCBI with accession numbers NP_001016403 (a) and NP_001164667 (b).
- Homologs from the frog *Xenopus laevis* were obtained from UniProt with accession numbers Q6DE61 (a), Q90WN3 (a), and Q7ZWV0 (b).
- Homologs from the fish medaka (*Oryzias latipes*) were obtained from Ensembl (Assembly: HdrR, Oct 2005. Genebuild Ensembl: Oct 2006) with identifiers ENSORLP00000008832 (a), ENSORLP00000018527 (a), ENSORLP00000007943 (b), and ENSORLP00000021647 (b). Computationally predicted exon 1 of ENSORLP00000018527 is very likely to be incorrect and was removed.
- Homologs from stickleback (*Gasterosteus aculeatus*) were obtained from Ensembl (Assembly: BROAD S1, Feb 2006. Genebuild Ensembl: Aug 2006) with identifiers ENSGACP00000013822 (a), ENSGACP00000024909 (a), ENSGACP00000023579 (b), and ENSGACP00000012693 (b). Splicing of intron 5 was corrected in ENSGACP00000024909.
- Homologs from the fish tetraodon (*Tetraodon nigroviridis*) were obtained from Ensembl (Assembly: TETRAODON 8.0, Mar 2007. Genebuild Ensembl: Jul 2008) with identifiers ENSTNIP00000013350 (a), ENSTNIP00000015136 (b), and ENSTNIP00000011358 (b).
- Homologs from the fish fugu (*Takifugu rubripes*) were obtained from Ensembl (FUGU 4.0, Jun 2005. Genebuild Ensembl: Mar 2008) with identifiers ENSTRUP00000013710 (a), ENSTRUP00000032221 (a), ENSTRUP00000015108 (b), and ENSTRUP00000026750 (b).
- Homologs from zebrafish (*Danio rerio*) were obtained from UniProt and the NCBI with accession numbers A3KMS9 (a), NP_001003470 (a), Q7T374 (b), and Q3ZB92 (b).
- Homologs from salmon (*Salmo salar*) were obtained from UniProt with the accession numbers C0H990 (b) and C0HBG9 (b).
- A homolog from *Ciona intestinalis* was obtained from Ensembl (Assembly: JGI 2, Mar 2005. Genebuild Ensembl: Feb 2006) with identifier ENSCINP00000013406.
- A homolog from *Ciona savignyi* was generated by using data from the Ensembl project (Assembly: CSAV 2.0, Oct 2005. Genebuild Ensembl: Jun 2006). The splicing of intron 9 in ENSCSAVP00000004909 was corrected in order to get better agreement with the other chordate homologs and the 4 nt intron 10 was removed based on evidence in NCBI trace archive (http://www.ncbi.nlm.nih.gov/Traces) entries with TI numbers 80807213 and 76035921.
- Homologs from sea urchin (*Strongylocentrotus purpuratus*) and amphioxus (*Branchiostoma floridae*) were obtained from the NCBI with accession numbers XP_001175934 and XP_002600447, respectively.
- The expressed sequence tag (EST) sequence FD727107 from sea lamprey (*Petromyzon marinus*) was obtained from the NCBI EST database. It corresponds to human Cα1/Cβ1 exons 2 to 8 and approximately half of exon 9. Several of these exons matched perfectly predicted exons found in genomic Contig3574 of the Pre!Ensembl assembly (http://pre.ensembl.org; Assembly: PMAR3, May 2007) for this organism. Two predicted exons matching full-length exon 9 and exon 10 were also detected in the same contig. A sea lamprey Cα/Cβ homolog comprising exons 2-10 was generated by combining the EST sequence with the exons predicted from genomic sequence. Importantly, all exons are 100% identical to segments in a number of whole genome shotgun (WGS) traces available from the NCBI trace archive, strongly supporting the correctness of this sequence.

A second sea lamprey Cα/Cβ homolog was built from predicted introns in Contig24197 of the Pre!Ensembl assembly. Only exons 2-6 and 8 were available for this homolog. Also in this case all exons are 100% identical to exonic segments in a number of WGS traces available from the NCBI trace archive.

- EST sequences from the NCBI databases from dogfish shark (*Squalus acanthias*) EE721729, CX790175, CX790155, and ES324818, were combined to give partial sequences for two shark Cα/Cβ homologs.
- EST sequences from the NCBI databases from little skate (*Leucoraja erinacea*) FL669520, CO050387, CO051416, CV221988, and GH547261 were combined to give partial sequences for two skate Cα/Cβ homologs.
- A homolog from the hemichordate *Saccoglossus kowalevskii* was obtained from the NCBI with accession number XP_002740161.
- A single homolog from the species fruit fly (*Drosophila melanogaster*), yellow fever mosquito (*Aedes aegypti*), red flour beetle (*Tribolium castaneum*), honey bee (*Apis mellifera*), and bont tick (*Amblyomma hebraeum*) were obtained from the NCBI with accession numbers NP_476977, XP_001652671, XP_968170, XP_393285, and CAG44453, respectively.
- Homologs from the mollusks great pond snail (*Lymnaea stagnalis*) and California sea hare (*Aplysia californica*) were obtained from UniProt with accession numbers Q4JIV3 and Q16957, respectively.
- Homologs from the nematode *Caenorhabditis elegans*, the sponge *Amphimedon queenslandica*, and the crustacean *Daphnia pulex* were obtained with NCBI accession numbers NP_740958, XP_003388521, and EFX78769, respectively.
